# Brain-derived neurotrophic factor from microglia regulates neuronal development in the medial prefrontal cortex and its associated social behavior

**DOI:** 10.1038/s41380-024-02413-y

**Published:** 2024-01-19

**Authors:** Takashi Komori, Kazuya Okamura, Minobu Ikehara, Kazuhiko Yamamuro, Nozomi Endo, Kazuki Okumura, Takahira Yamauchi, Daisuke Ikawa, Noriko Ouji-Sageshima, Michihiro Toritsuka, Ryohei Takada, Yoshinori Kayashima, Rio Ishida, Yuki Mori, Kohei Kamikawa, Yuki Noriyama, Yuki Nishi, Toshihiro Ito, Yasuhiko Saito, Mayumi Nishi, Toshifumi Kishimoto, Kenji F. Tanaka, Noboru Hiroi, Manabu Makinodan

**Affiliations:** 1 Department of Psychiatry, Nara Medical University, Kashihara, Nara 634-8521 Japan; 2 Department of Anatomy and Cell Biology, Nara Medical University, Kashihara, Nara 634-8521 Japan; 3 Department of Immunology, Nara Medical University, Kashihara, Nara 634-8521 Japan; 4 Department of Neurophysiology, Nara Medical University, Kashihara, Nara 634-8521 Japan; 5 Division of Brain Sciences, Institute for Advanced Medical Research, Keio University School of Medicine, Tokyo, 160-8582 Japan; 6 Department of Pharmacology, UT Health San Antonio, San Antonio, TX 78229 USA; 7 Department of Cellular and Integrative Physiology, UT Health San Antonio, San Antonio, TX 78229 USA; 8 Department of Cell Systems and Anatomy, UT Health San Antonio, San Antonio, TX 78229 USA

**Keywords:** Neuroscience, Molecular biology

## Abstract

Microglia and brain-derived neurotrophic factor (BDNF) are essential for the neuroplasticity that characterizes critical developmental periods. The experience-dependent development of social behaviors—associated with the medial prefrontal cortex (mPFC)—has a critical period during the juvenile period in mice. However, whether microglia and BDNF affect social development remains unclear. Herein, we aimed to elucidate the effects of microglia-derived BDNF on social behaviors and mPFC development. Mice that underwent social isolation during p21–p35 had increased *Bdnf* in the microglia accompanied by reduced adulthood sociability. Additionally, transgenic mice overexpressing microglial *Bdnf*—regulated using doxycycline at different time points—underwent behavioral, electrophysiological, and gene expression analyses. In these mice, long-term overexpression of microglial BDNF impaired sociability and excessive mPFC inhibitory neuronal circuit activity. However, administering doxycycline to normalize BDNF from p21 normalized sociability and electrophysiological function in the mPFC, whereas normalizing BDNF from later ages (p45–p50) did not normalize electrophysiological abnormalities in the mPFC, despite the improved sociability. To evaluate the possible role of BDNF in human sociability, we analyzed the relationship between adverse childhood experiences and *BDNF* expression in human macrophages, a possible proxy for microglia. Results show that adverse childhood experiences positively correlated with *BDNF* expression in M2 but not M1 macrophages. In summary, our study demonstrated the influence of microglial BDNF on the development of experience-dependent social behaviors in mice, emphasizing its specific impact on the maturation of mPFC function, particularly during the juvenile period. Furthermore, our results propose a translational implication by suggesting a potential link between BDNF secretion from macrophages and childhood experiences in humans.

## Introduction

Microglia refine synapses and form developing brain circuits in an activity-dependent manner [1, 2]. This process is essential for normal brain development [3]. A limited critical postnatal period of development exists in the sensory cortexes of mice, during which neural network plasticity is transiently enhanced, and the experience-dependent flexible reorganization of neural circuits occurs [4, 5]. Microglia are involved in activity-dependent brain formation processes during brain development [1–3, 6, 7]. Notably, recent studies suggest that microglia may be actively involved in the critical-period plasticity [8–10].

Furthermore, similar to sensory functions, social behaviors develop via experience-dependent brain maturation during a limited postnatal window [11, 12]. For instance, mice that undergo social isolation 21–35 days postnatal (p21–p35) later display social impairment that is irreversible by re-socialization [12]. Juvenile social deprivation harms the neural circuits and glial cells of the medial prefrontal cortex (mPFC) [11–18], one of the regions regulating social behaviors [19]. Previously, we reported the involvement of microglia in juvenile social isolation and future social impairment [13]. Meanwhile, others have implicated microglia in social development [20–22]. Additionally, microglia play a role in the synaptic pruning of the prefrontal cortex [23] and in the time-specific functional maturation of mPFC in rodents [24]. These findings suggest that microglia are involved in mPFC maturation and its associated sociability function in a time-specific manner via activity-dependent neuroplasticity and pruning.

Brain-derived neurotrophic factor (BDNF) contributes to maturing inhibitory interneurons and closing critical periods [25]. Microglia secrete BDNF [26–28], which is relevant for learning-dependent synaptic plasticity in the motor cortex [29] and its effect on inhibitory synaptic transmission in the spinal cord [26]. However, it remains unclear whether microglia-secreted BDNF (MG-BDNF) influences the cortical critical-period plasticity and behavior [8, 30]. In this study, we investigate the time-specific effects of microglia on mPFC biology and social behavior, focusing on MG-BDNF.

## Materials and methods

### Animals

All study protocols were approved by the Animal Care Committee of Nara Medical University in accordance with the policies established in the National Institutes of Health Guide for the Care and Use of Laboratory Animals. The animals were housed in a temperature- and humidity-controlled animal facility under a standard 12-h light-dark cycle (lights on 08:00–20:00) with *ad libitum* access to food and water throughout the study. C57BL/6 J mice isolated from the weaning age (p21) for two weeks were regrouped with age-, sex-, and strain-matched mice at p35 (3–5 mice per cage), labeled juvenile social isolation (j-SI) mice. Control C57BL6/J mice were group-housed following weaning at p21 and labeled group-housed (GH) mice. We crossed ionized calcium-binding adapter molecule 1 (*Iba1*) promoter driving tetracycline transactivator (*Iba1*-tTA) transgenic mice (line 75) [31] with tetracycline operator-*Bdnf* knock-in homozygous (*Bdnf* ^(tetO/tetO)^) mice [32, 33] and obtained microglia-specific *Bdnf*-overexpressing mice (*Iba1*-tTA::*Bdnf* ^(tetO/+)^ mice) and control mice (*Bdnf* ^(tetO/+)^ mice). Both *Iba1*-tTA and *Bdnf* ^(tetO/tetO)^ mice strains had a mixed C57BL/6 J and 129/SvEv background; only F1 male mice were used for all experiments [34]. Genotyping was confirmed as in previous studies [31, 32]. To temporally control *Bdnf* overexpression, F1 mice were fed doxycycline (DOX)-containing chow (DOX 100 mg/kg, CLEA Japan Inc., Tokyo, Japan): from weaning (p21) or adulthood (p45–p50). Groups of mice administered DOX from adulthood were provided chow with DOX *ad libitum* for at least two weeks before the experiment. In all studies, subjects were randomly chosen from all litters.

### Statistical analysis

All statistical analyses were performed using Prism v.9 (GraphPad Software Inc., San Diego, CA, USA). The sample size was determined based on our prior studies [12–14]. When the sample data were normally distributed and had equal variances, as determined using the Shapiro–Wilk test and the subsequent F-test, significant differences between groups were assessed using an unpaired two-tailed Student’s *t* test. Even if the sample data passed the Shapiro–Wilk test for normality, the nonparametric Mann–Whitney *U*-test was used if the F-test indicated unequal variances. When the sample data significantly differed from normal distribution as assessed by the Shapiro–Wilk test, the nonparametric Mann–Whitney *U*-test was used. Values with right-skewed distribution were log-transformed for statistical analyses. The Kruskal–Wallis test was used to validate microglia-specific BDNF-overexpressing levels. Two-way ANOVA followed by a Bonferroni post hoc test were used to analyze the time spent in each zone in the three-chamber social preference test [35]. Two-way repeated measures ANOVA was used to assess the cumulative number of approaches in the augmented reality-based long-term animal behavior observing system. Spearman’s rank correlation coefficient was used to evaluate correlations between *Bdnf* expression in peripheral blood mononuclear cells and microglia in mice, as well as between the Child Abuse and Trauma Scale (CATS) scores and *BDNF* in macrophages in humans. The false discovery rate-controlling Benjamini–Hochberg procedure was used to control for multiple comparisons [36]. All data were presented as the mean ± standard error of the mean (SEM); *p* values < 0.05 were considered statistically significant. The q-values were determined for multiple comparisons, and values < *q* value were significant.

Detailed methodological information is provided in the Supplementary materials and methods.

## Results

### Juvenile social isolation (j-SI) mice are socially impaired and have increased MG-*Bdnf*

**Fig. 1:**
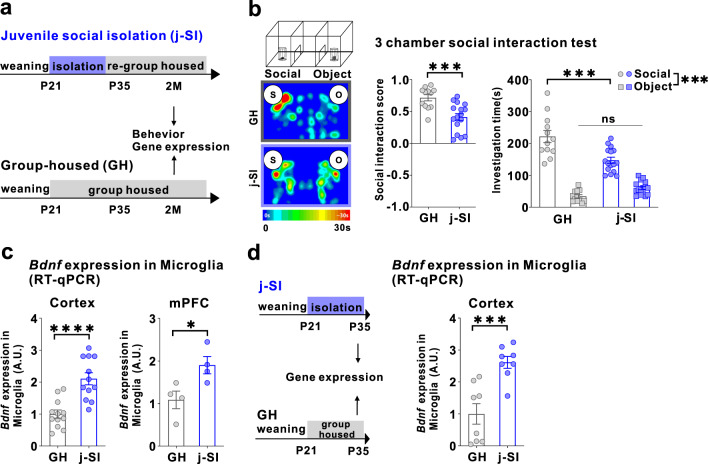
Juvenile social isolation reduces sociability and increases MG-*Bdnf* expression. **a** Schema of the group-housed (GH) and juvenile social isolation (j-SI) mice time series. The experiments started at p56. **b** j-SI mice displayed reduced sociability in the three-chamber sociability test compared with GH mice. j-SI mice had a lower social interaction score than GH mice (t_(26)_ = 3.948, *p* = 0.0005, unpaired two-tailed Student’s *t* test, GH: *n* = 12, j-SI: *n* = 16) and spent less time near novel mice (F_1,26_ (interaction) = 17.16, *p* = 0.0003, two-way ANOVA followed by Bonferroni post hoc test, Bonferroni post hoc analysis of GH S vs. j-SI S, *p* < 0.0001 GH: *n* = 12, j-SI: *n* = 16). S, social; O, object. **c** (Left) *Bdnf* expression measured using real-time quantitative polymerase chain reaction (RT-qPCR) in microglia recovered from the cortex was higher in the j-SI mice than in the GH mice (t_(22)_ = 4.876, *p* < 0.0001, unpaired two-tailed Student’s *t* test, GH: *n* = 12, j-SI: *n* = 12). (Right) *Bdnf* expression measured using RT-qPCR in microglia recovered from the medial prefrontal cortex (mPFC) was higher in j-SI than in GH mice (t_(6)_ = 2.818, *p* = 0.0304, unpaired two-tailed Student’s *t* test, GH: *n* = 4 (from 16 mice), j-SI: *n* = 4 (from 16 mice)). The value was log-transformed with a base of 10 because of the right-skewed distribution. Each dot indicates the expression of *Bdnf* in microglia collected from the mPFC of four mice. **d** (Left) Schema of the GH and j-SI mice time series. The experiments started at P35. (right) *Bdnf* expression measured using RT-qPCR in microglia recovered from the cortex at P35 was higher in the j-SI mice than in the GH mice (t(14) = 4.353, *p* = 0.0007, unpaired two-tailed Student’s *t* test, GH: *n* = 8, j-SI: *n* = 8). *p  <  0.05, ***p  <  0.001, ****p  <  0.0001. Data are presented as the mean ± SEM. 2 M: two months of age, A.U.: arbitrary unit.

Our single housing condition started at p21 and lasted for two weeks, followed by re-grouped housing with age-, sex-, and strain-matched mice (Fig. 1a). While single housing conditions induce varying alterations in the brain and behavioral consequences in mice, depending on the strain, sex, and age [37, 38], our procedure has been shown to induce agitation and defective social behaviors and alter the plasticity in the mPFC [12, 14, 15, 39, 40]. In this study, C57BL/6 J male mice isolated during p21–p35 also exhibited impaired sociability in a three-chamber social preference test at two months of age (Fig. 1a, b). In the open field test, no differences in basal activity or time spent in the center existed between j-SI and GH mice (Supplementary Fig. 1a, b). *Bdnf* mRNA expression in microglia from the cerebral and prefrontal cortexes was higher in j-SI mice than GH mice (Fig. 1c). However, no differences were observed in *Bdnf* mRNA expression in the bulk tissues of the cerebral cortex and mPFC between the j-SI and GH mice (Supplementary Fig. 1a, c). These findings indicate that microglial *Bdnf* expression changes may be specific to microglia [29]. To elucidate the impact of the absence of social stimulation during the juvenile period or subsequent re-socialization on microglial *Bdnf* mRNA expression in the cerebral cortex, we measured *Bdnf* mRNA expression in microglia from the cerebral cortex of GH and j-SI mice at p35. Notably, by p35, the end of j-SI, *Bdnf* mRNA expression in microglia in j-SI mice was already significantly higher than that in GH mice (Fig. 1d). These results suggest that j-SI experiences induce *Bdnf* mRNA overexpression in microglia, persisting into adulthood even after re-socialization, with no improvement.

### Persistently overexpressed MG-BDNF causes social impairment and functional changes in the mPFC pyramidal cells

**Fig. 2:**
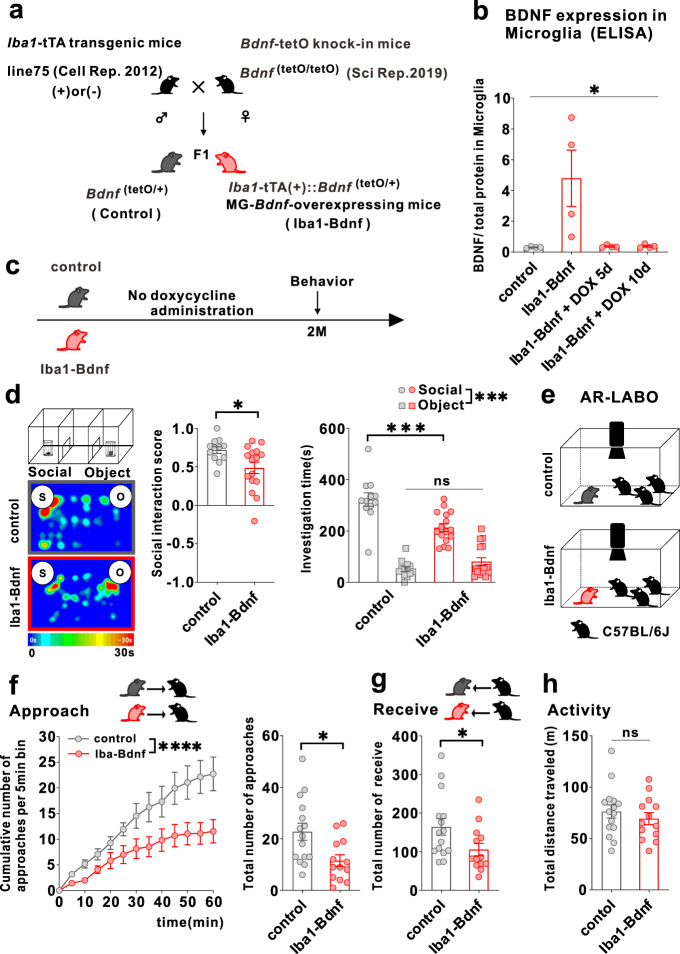
Overexpression of MG-BDNF leads to impaired sociability in adulthood. **a** Diagram of the generation of MG-*Bdnf*-overexpressing mice. The tet-off system was activated in double transgenic mice (F1), which were obtained by crossing *Iba1*-tTA transgenic mice with *Bdnf*-tetO knock-in mice. *Iba1*-tTA(+)::*Bdnf*^(tetO/+)^ mice were the MG-*Bdnf*-overexpressing mice, and *Bdnf*^(tetO/+)^ mice were the control mice. **b** BDNF expression in microglia recovered from the cortex, as measured using ELISA. *Iba1*-tTA(+)::*Bdnf*^(tetO/+)^ mice had higher BDNF expression than *Bdnf*^(tetO/+)^ mice; this was normalized from day 5 using doxycycline (DOX) (The Kruskal-Wallis test, 9.086, *p* = 0.0118; *n* = 4 per group). **c** Schema of the behavioral experiments without doxycycline. The experiments were started at P61. **d** The *Iba1*-tTA(+)::*Bdnf*^(tetO/+)^ mice were less social in the three-chamber sociability test than the *Bdnf*^(tetO/+)^ mice. The *Iba1*-tTA(+)::*Bdnf*^(tetO/+)^ mice had a lower social interaction score than *Bdnf*^(tetO/+)^ mice (U = 47, *p* = 0.0226, Mann–Whitney U test, *Bdnf*^(tetO/+)^: *n* = 12, *Iba*1-tTA(+)::*Bdnf*^(tetO/+)^: *n* = 16) (left) and spent less time near novel mice (F_1,26_(interaction) = 14.63, *p* = 0.0007, two-way ANOVA followed by Bonferroni post hoc test; Bonferroni post hoc analysis of *Iba1*-tTA(+)::*Bdnf*^(tetO/+)^ S vs. *Bdnf*^(tetO/+)^ S, *p* = 0.0001, *Bdnf*^(tetO/+)^: *n* = 12, *Iba1*-tTA(+)::*Bdnf*^(tetO/+)^: *n* = 16) (right). S, social; O, object. **e** Schematic of the Augmented Reality-based Long-term Animal Behavior Observing system (AR-LABO). Three novel C57BL/6 J mice were placed with a target mouse and observed for 1 h. **f** (Left) The cumulative number of approaches per 5-min bin for *Bdnf*^(tetO/+)^ and *Iba1*-tTA(+)::*Bdnf*^(tetO/+)^ mice. *Iba1*-tTA(+)::*Bdnf*^(tetO/+)^ mice accumulates significantly less cumulative approaches to other mice than *Bdnf*^(tetO/+)^ mice (two-way RM ANOVA, phenotype (control or Iba1-BDNF) × time (5 min bin) interaction F_(12,312)_  =  3.926, *p*  < 0.0001; effect of phenotype F_(1,26)_  =  6.670, *p*  =  0.0158; effect of time F_(12,312)_  =  39.31, *p*  < 0.0001, *Bdnf*^(tetO/+)^: *n* = 15, *Iba1*-tTA(+)::*Bdnf*^(tetO/+)^: *n* = 13). (Right) *Iba1*-tTA(+)::*Bdnf*^(tetO/+)^ mice approached other mice less than the *Bdnf*^(tetO/+)^ mice (t_(26)_ = 2.717, *p* = 0.0116, unpaired two-tailed Student’s *t* test, *Bdnf*^(tetO/+)^: *n* = 15, *Iba1*-tTA(+)::*Bdnf*^(tetO/+)^: *n* = 13). **g** The *Iba1*-tTA(+)::*Bdnf*^(tetO/+)^ mice were less approached by other mice than *Bdnf*^(tetO/+)^ mice (t_(26)_ = 2.120, *p* = 0.0437, unpaired two-tailed Student’s *t* test, *Bdnf*^(tetO/+)^: *n* = 15, *Iba1*-tTA(+)::*Bdnf*^(tetO/+)^: *n* = 13). **h** No differences in activity were observed during AR-LABO between the *Iba1*-tTA(+)::*Bdnf*^(tetO/+)^ and *Bdnf*^(tetO/+)^ mice (t_(26)_ = 0.8059, *p* = 0.4276, unpaired two-tailed Student’s *t* test, *Bdnf*^(tetO/+)^: *n* = 15, *Iba1*-tTA(+)::*Bdnf*^(tetO/+)^: *n* = 13). *p  <  0.05, ***p  <  0.001. Data are presented as the mean ± SEM. 2 M: two months of age, control: *Bdnf*^(tetO/+)^ mice, Iba1-Bdnf: *Iba1*-tTA(+)::*Bdnf*^(tetO/+)^ mice.

To recapitulate the microglia-specific and sustained *Bdnf* overexpression observed in j-SI and examine the causal relationship between microglia-specific *Bdnf* gene upregulation and social impairment, we exploited a tet-off system [41] to induce microglia-specific *Bdnf* mRNA expression (Fig. 2a). MG-BDNF overexpression was confirmed in *Iba1*-tTA::*Bdnf*^(tetO/+)^ mice without DOX and MG-BDNF overexpression in *Iba1*-tTA::*Bdnf*^(tetO/+)^ mice was normalized from day 5 after orally administering DOX (Fig. 2b). *Bdnf* mRNA expression in the bulk tissues of the cerebral cortex and mPFC did not differ between the *Iba1*-tTA::*Bdnf*^(tetO/+)^ and control mice, suggesting that genetically-induced MG-*Bdnf* overexpression in *Iba1*-tTA::*Bdnf*^(tetO/+)^ mice does not significantly alter total *Bdnf* mRNA expression levels in the cerebral cortex or mPFC (Supplementary Fig. 2a, b).

Next, we evaluated whether persistent MG-BDNF overexpression impairs social behavior at two months of age, such as in j-SI mice, using a three-chamber social preference test (Fig. 2c). The *Iba1*-tTA::*Bdnf*^(tetO/+)^ mice had a lower social interaction score than the control mice and spent less time around the novel mice (Fig. 2d). In contrast, no differences in locomotor activity or time spent in the center existed between the *Iba1*-tTA::*Bdnf*^(tetO/+)^ and control mice (Supplementary Fig. 2a, c). However, considering that the three-chamber apparatus has certain interpretative issues [42] and that social behavior through a barrier has different molecular substrates than true physical social interactions [43], we further assessed real social behavior in a naturalistic environment among multiple animals using the augmented reality-based long-term animal behavior observing system (AR-LABO). This system is an improved version of our previous system that can determine the position of each mouse under social housing conditions [44–46]. The subject mice and three age-matched male C57BL/6 J mice that had never been cohoused with each other were placed in a new cage at two months of age, and their movements were observed for 1 h (Fig. 2e). Compared to the control, the *Iba1*-tTA::*Bdnf*^(tetO/+)^ mice made fewer approaches to other mice (Fig. 2f). They were also less often approached by other mice (Fig. 2g). No difference in total activity existed between the *Iba1*-tTA::*Bdnf*^(tetO/+)^ and control mice during the 1-h recording time (Fig. 2h). These results suggest that sustained MG-BDNF overexpression induces social deficits in adulthood.

**Fig. 3:**
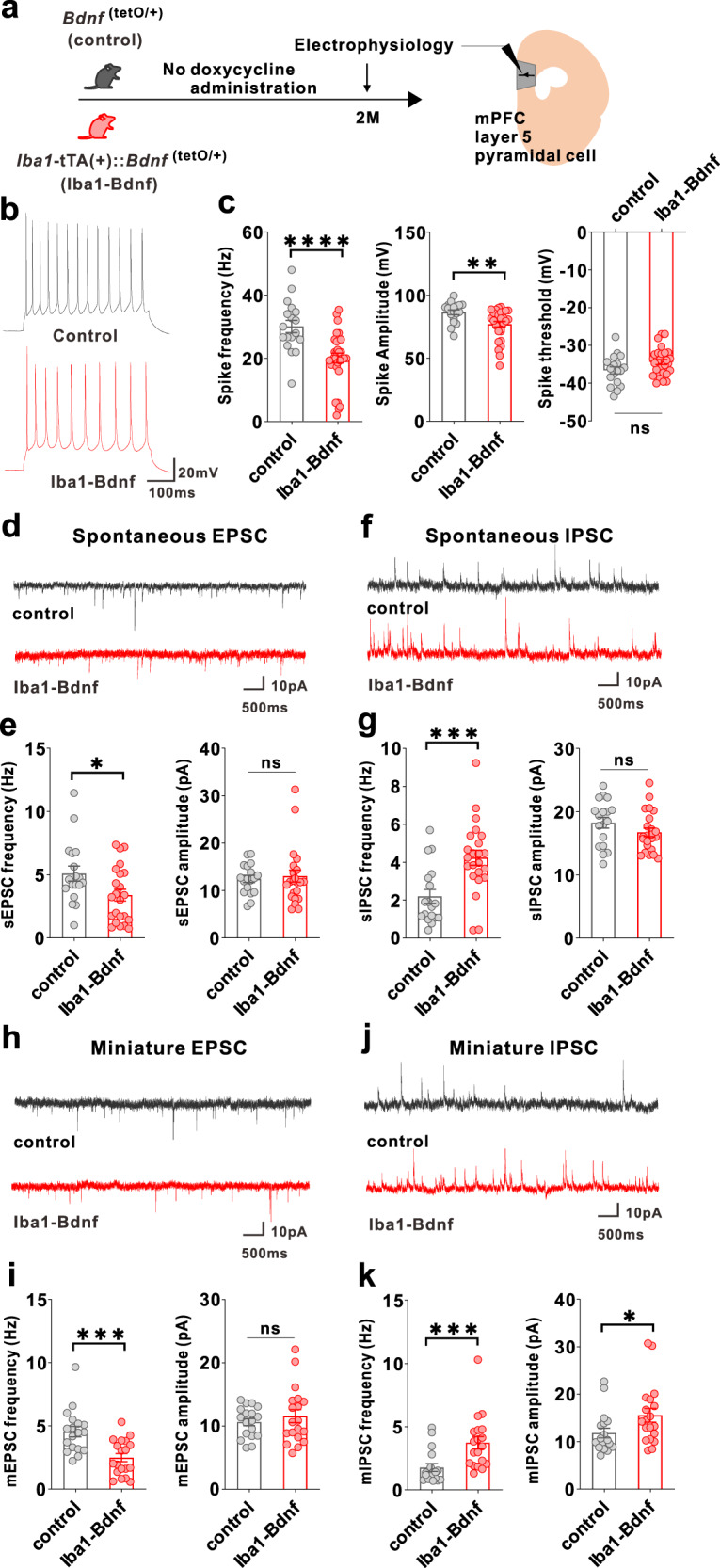
MG-BDNF overexpression affects the inhibitory synaptic inputs to mPFC pyramidal cells and the excitability of such cells. **a** Electrophysiological analysis of mPFC layer V pyramidal cells in adulthood without doxycycline administration. The experiments were started at P62. **b** Representative traces recorded from mPFC pyramidal cells at 200-pA injection. **c** *Iba1*-tTA(+)::*Bdnf*^(tetO/+)^ mice had lower spike frequency (U = 93.50, *p* < 0.0001, Mann–Whitney U test) (left) and amplitude (U = 131, *p* = 0.0026, Mann–Whitney U test) (middle) than *Bdnf*^(tetO/+)^ mice at 200-pA injection. No significant differences existed in spike threshold (t_(46)_ = 1.997, *p* = 0.0518, unpaired two-tailed Student’s *t* test) (right). (*n*  =  18 cells from three biologically independent *Bdnf*^(tetO/+)^ mice, *n*  =  30 cells from four biologically independent *Iba1*-tTA(+)::*Bdnf*^(tetO/+)^ mice). **d** Representative traces of spontaneous excitatory postsynaptic currents (sEPSCs). **e** (Left) The *Iba1*-tTA(+)::*Bdnf*^(tetO/+)^ mice had lower sEPSC frequency than *Bdnf*^(tetO/+)^ mice (U = 130, *p* = 0.0436, Mann–Whitney U test). (Right) No significant difference existed in sEPSC amplitude (U = 185, *p* = 0.5761, Mann–Whitney U test). **f** Representative traces of spontaneous inhibitory postsynaptic currents (IPSCs). **g** (Left) *Iba1*-tTA(+)::*Bdnf*^(tetO/+)^ mice showed increased sIPSC frequency compared with *Bdnf*^(tetO/+)^ mice (U = 85, *p* = 0.0010, Mann–Whitney U test). (Right) There was no significant difference in sIPSC amplitude (t_(39)_ = 1.440, *p* = 0.1579, unpaired two-tailed Student’s *t* test). **e**, **g** *n*  =  18 cells from three biologically independent *Bdnf*^(tetO/+)^ mice, *n*  =  23 cells from four biologically independent *Iba1*-tTA(+)::*Bdnf*^(tetO/+)^ mice. **h** Representative traces of miniature EPSCs (mEPSCs). **i** (Left) *Iba1*-tTA(+)::*Bdnf*^(tetO/+)^ mice had lower mEPSC frequency than *Bdnf*^(tetO/+)^ mice. (t_(35)_ = 3.949, *p* = 0.0004, unpaired two-tailed Student’s *t* test). (Right) There was no significant difference in mEPSC amplitude (U = 158, *p* = 0.7074, Mann–Whitney U test). **j** Representative traces of miniature IPSCs (mIPSCs). (k) The *Iba1*-tTA(+)::*Bdnf*^(tetO/+)^ mice had increased mIPSC frequency (U = 53, *p* = 0.0002, Mann–Whitney U test) (left) and amplitude (U = 101, *p* = 0.0335, Mann–Whitney U test) (right) than the *Bdnf*^(tetI/+)^ mice. **i**, **k** *n*  =  18 cells from three biologically independent *Bdnf*^(tetO/+)^ mice, *n*  = 19 cells from three biologically independent *Iba1*-tTA(+)::*Bdnf*^(tetO/+)^ mice. *p  <  0.05, **p  <  0.01, ***p  <  0.001, ****p  <  0.0001. Data are presented as the mean ± SEM. 2 M: two months of age, control: *Bdnf*^(tetO/+)^ mice, Iba1-Bdnf: *Iba1*-tTA(+)::*Bdnf*^(tetO/+)^ mice.

To address whether artificial BDNF overexpression causes dysfunction in mPFC layer V pyramidal cells, as demonstrated by our previous research where j-SI mice displayed this dysfunction [16, 17], we examined these cells using whole-cell patch-clamp recordings at two months of age (Fig. 3a). Compared with the adult control mice, excitability was significantly reduced in the adult *Iba1*-tTA::*Bdnf*^(tetO/+)^ mice in terms of spike frequency and amplitude (Fig. 3b, c). In contrast, no significant differences existed in the spike thresholds (Fig. 3c). Spontaneous excitatory postsynaptic current (sEPSC) frequency was significantly lower (Fig. 3d, e), and spontaneous inhibitory postsynaptic current (sIPSC) frequency was significantly higher (Fig. 3f, g) in the adult *Iba1*-tTA::*Bdnf*^(tetO/+)^ mice compared with the control mice. In contrast, sEPSC and sIPSC amplitudes did not significantly differ (Fig. 3e, g). Furthermore, in *Iba1*-tTA::*Bdnf*^(tetO/+)^ mice, miniature EPSC (mEPSC) frequency was significantly lower (Fig. 3h, i), and miniature IPSC (mIPSC) frequency and amplitude were significantly higher than in the control mice (Fig. 3j, k). These results suggest that a sustained increase in MG-BDNF from early childhood may decrease the excitability of mPFC layer V pyramidal cells. It also increases the number of inhibitory synaptic inputs and decreases the excitatory synaptic inputs in such cells, enhancing the activity of inhibitory neuronal circuits. These abnormal mPFC changes were similar to those observed in j-SI mice in our previous study [16, 17]. To confirm the electrophysiological impact of MG-BDNF overexpression in other brain regions, we conducted whole-cell patch-clamp recordings in the posterior paraventricular thalamus (pPVT) of *Iba1*-tTA::*Bdnf*^(tetO/+)^ and control mice, where no abnormalities were observed in j-SI mice in previous research [14]. There were no significant differences in excitatory and inhibitory inputs to pPVT between the *Iba1*-tTA::*Bdnf*^(tetO/+)^ and control mice (Supplementary Fig. 2d, e, f, g), consistent with the findings in j-SI mice [14]. In summary, our results demonstrated that overexpressed MG-BDNF was sufficient to induce mPFC dysfunction and social impairment.

### MG-BDNF affects the complement system in the mPFC

**Fig. 4:**
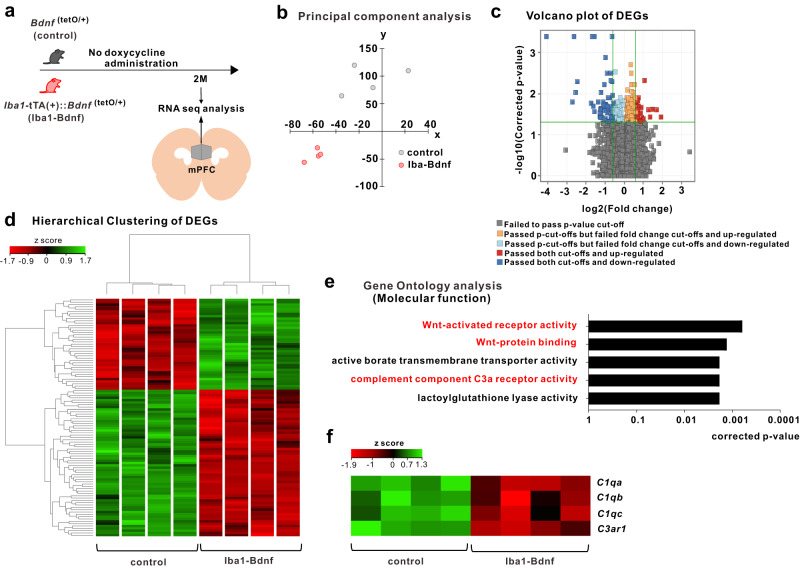
MG-BDNF overexpression affects the complement system. **a** RNA-seq analysis of the mPFC in adult *Bdnf*^(tetO/+)^ (*n* = 4) and *Iba1*-tTA(+)::*Bdnf*^(tetO/+)^ (*n* = 4) mice without doxycycline. The experiments were started at P64. **b** Principal component analysis revealing gene expression differences between the *Bdnf*^(tetO/+)^ and *Iba1*-tTA(+)::*Bdnf*^(tetO/+)^ mice. **c** Volcano plot of differentially expressed genes (DEGs). The thresholds are log_2_ fold change >1.5 and *p* < 0.05. **d** Heatmap and hierarchical clustering of DEGs between *Bdnf*^(tetO/+)^ and *Iba1*-tTA(+)::*Bdnf*^(tetO/+)^ mice. The thresholds are log_2_ fold change >1.5 and *p* < 0.05. Gene expression levels are indicated in the heatmap by the Z-scores in the legend. **e** The Gene Ontology analysis of down-regulated DEGs in the *Iba1*-tTA(+)::*Bdnf*^(tetO/+)^ mice compared with *Bdnf*^(tetO/+)^ mice suggested the involvement of the Wnt signaling pathway (Wnt-activated receptor pathway, *p* = 0.0006; Wnt-protein binding, *p* = 0.0013) and complement component C3a receptor activity (*p* = 0.0018) in molecular function. **f** Differences in expression levels of selected complement genes in *Bdnf*^(tetO/+)^ and *Iba1*-tTA(+)::*Bdnf*^(tetO/+)^ mice in RNA-Seq analysis. Gene expression levels are presented in the heatmap by the Z-scores in the legend. 2 M: two months of age, control: *Bdnf*^(tetO/+)^ mice, Iba1-Bdnf: *Iba1*-tTA(+)::*Bdnf*^(tetO/+)^ mice.

RNA-sequencing and principal component analysis revealed that MG-BDNF overexpression during the juvenile period altered mPFC distribution at two months of age (Fig. 4a, b). Compared with control mice, 63 genes were downregulated and 39 were upregulated in the adult *Iba1*-tTA::*Bdnf*^(tetO/+)^ mice (Fig. 4c, d). Gene Ontology molecular function analysis using differentially expressed genes downregulated in *Iba1*-tTA::*Bdnf*^(tetO/+)^ mice revealed that sustained MG-BDNF overexpression altered gene expression related to Wnt-activated receptor activity (corrected *p* value < 0.0001), Wnt-protein binding (corrected *p* value < 0.0001), active borate transmembrane transporter activity (corrected *p* value = 0.0018), and complement C3a receptor activity (corrected *p* value = 0.0018; Fig. 4e). Heatmaps of Wnt signaling- and complement system-related gene expression were indicated with Z-scores, referencing the Kyoto Encyclopedia of Genes and Genomes pathways [47] (Supplementary Fig. 3a and Fig. 4f). This heat mapping of *C1qa*, *C1qb*, *C1qc*, and *C3ar1* expression using Z-scores suggested reduced function of this complement cascade beginning with *C1q* in *Iba1*-tTA::*Bdnf*^(tetO/+)^ mice. In particular, the *Iba1*-tTA::*Bdnf*^(tetO/+)^ mice had significantly decreased *C1qa* (corrected *p* value = 0.0241) and *C3ar1* expression (corrected *p* value = 0.0288). We compared the levels of various neurotrophic factors and cytokines in the mPFC of *Iba1*-tTA::*Bdnf*^(tetO/+)^ and control mice, including nerve growth factor (*Ngf*), neurotrophin-3 and −5 (*Ntf3, Ntf5*), tumor necrosis factor (*Tnf*), interleukin (*IL*)-1β, *IL-6*, *IL-10*, *IL-12a*, glial cell line-derived neurotrophic factor (*Gdnf*), insulin-like growth factor 1 (*Igf1*), and transforming growth factor-β (*Tgf-β*). However, no significant differences were observed between these two groups (Supplementary Fig. 3b).

Furthermore, to investigate whether these changes were solely induced by the altered gene expression of complement system-related genes within microglia due to BDNF overexpression, we compared the expression of *C1qa*, *C1qb*, *C1qc*, and *C3ar1* in microglia recovered from the cortex of *Iba1*-tTA::*Bdnf*^(tetO/+)^ and control mice. However, no significant differences were observed (Supplementary Fig. 3c), suggesting that MG-BDNF overexpression does not drive complement system-related gene expression changes solely within microglia. In summary, our results suggest that MG-BDNF overexpression reduces the functional status of the complement cascade in the mPFC and that this effect is not solely attributed to MG-BDNF overexpression affecting complement system-related gene expression in microglia.

### Normalizing MG-BDNF overexpression during the juvenile period rescues impaired sociability and mPFC pyramidal cell dysfunction in adulthood

**Fig. 5:**
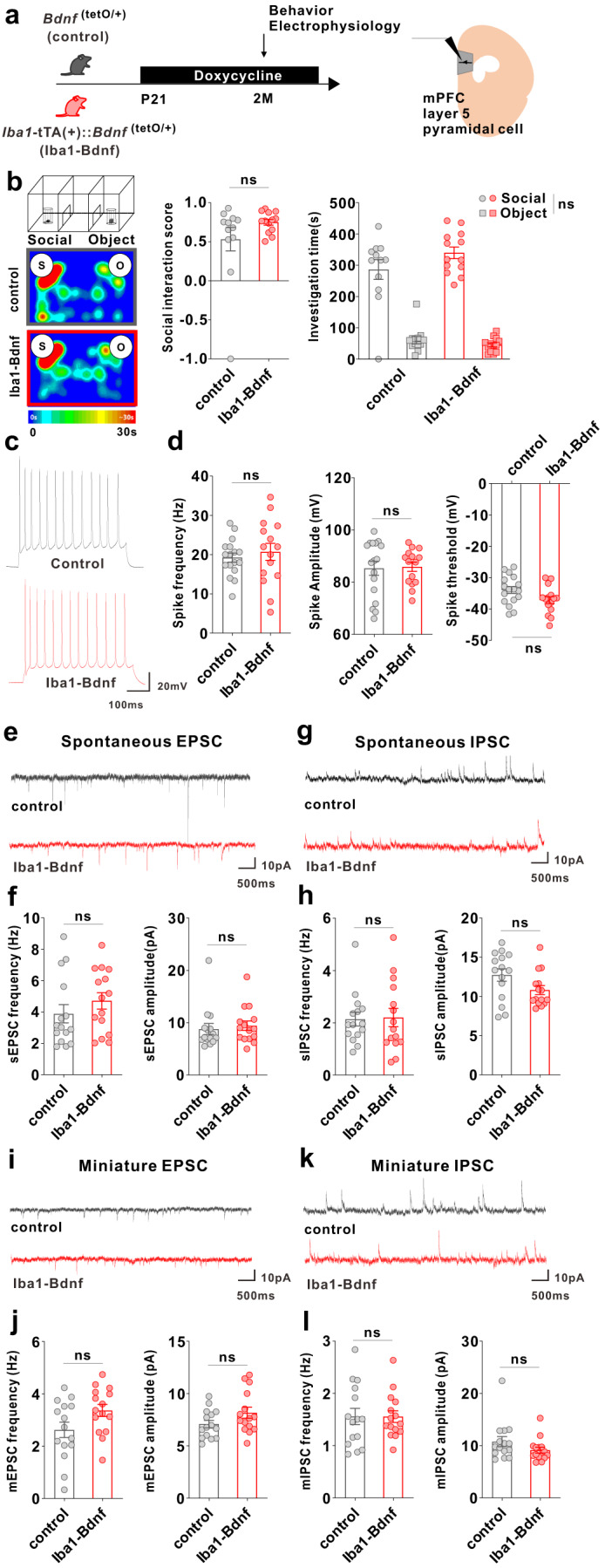
Normalizing MG-BDNF during the juvenile period does not impair sociability or abnormal inhibitory inputs in the mPFC. **a**–**l** The *Iba1*-tTA(+)::*Bdnf*^(tetO/+)^ mice were administered doxycycline from p21 at weaning to normalize MG-BDNF. *Bdnf*^(tetO/+)^ mice were also administered doxycycline from p21 as the control. Behavioral and electrophysiological experiments were started at p61. **b** Normalizing MG-BDNF from p21 did not reduce sociability in adult *Iba1*-tTA(+)::*Bdnf*^(tetO/+)^ mice in the three-chamber social test. Both groups had no differences in the social interaction score (U = 57, *p* = 0.2701, Mann–Whitney U test, *Bdnf*^(tetO/+)^: *n* = 12, *Iba1*-tTA(+)::*Bdnf*^(tetO/+)^: *n* = 13) (left) or social investigation time (F_1,23_(interaction) = 2.626, *p* = 0.1187, two-way ANOVA, *Bdnf*^(tetO/+)^: *n* = 12, *Iba1*-tTA(+)::*Bdnf*^(tetO/+)^: *n* = 13) (right). S, social; O, object. **c** Representative traces recorded from mPFC pyramidal cells at 200-pA injection. **d** No differences existed between *Bdnf*^(tetO/+)^ and *Iba1*-tTA(+)::*Bdnf*^(tetO/+)^ mice treated with doxycycline from p21 in the spike frequency (U = 110, *p* = 0.5192, Mann–Whitney U test) (left), spike amplitude (U = 120, *p* = 0.7944, Mann–Whitney U test) (middle), or threshold (t_(30)_ = 2.026, *p* = 0.0517, unpaired two-tailed Student’s *t* test) (right) at 200-pA injection (*n*  =  17 cells from three biologically independent *Bdnf*^(tetO/+)^ mice, *n*  =  15 cells from four biologically independent *Iba1*-tTA(+)::*Bdnf*^(tetO/+)^ mice). **e** Representative traces of spontaneous EPSCs. **f** No differences were observed between *Bdnf*^(tetO/+)^ and *Iba1*-tTA(+)::*Bdnf*^(tetO/+)^ mice treated with doxycycline from p21 in sEPSC frequency (U = 85, *p* = 0.2671, Mann–Whitney U test) (left) or amplitude (U = 89, *p* = 0.3453, Mann–Whitney U test) (right). **g** Representative traces of spontaneous IPSCs. (h) Normalizing MG-BDNF from p21 did not increase sIPSC frequency (U = 107, *p* = 0.8381, Mann–Whitney U test) (left) or amplitude (U = 69, *p* = 0.0742, Mann–Whitney U test) (right) in *Iba1*-tTA(+)::*Bdnf*^(tetO/+)^ mice. **f**, **h** *n*  =  15 cells from five biologically independent *Bdnf*^(tetO/+)^ mice, *n*  =  15 cells from three biologically independent *Iba1*-tTA(+)::*Bdnf*^(tetO/+)^ mice. **i** Representative traces of miniature EPSCs. **j** No differences existed between *Bdnf*^(tetO/+)^ and *Iba1*-tTA(+)::*Bdnf*^(tetO/+)^ mice treated with doxycycline from p21 in mEPSC frequency (t_(28)_ = 1.988, *p* = 0.0566, unpaired two-tailed Student’s *t* test) (left) or amplitude (t_(28)_ = 1.729, *p* = 0.0948, unpaired two-tailed Student’s *t* test) (right). **k** Representative traces of miniature IPSCs. **l** Normalizing MG-BDNF from p21 did not increase mIPSC frequency (t_(28)_ = 0.01783, *p* = 0.9859, unpaired two-tailed Student’s *t* test) (left) or amplitude (U = 76, *p* = 0.1370, Mann–Whitney U test) (right) in *Iba1*-tTA(+)::*Bdnf*^(tetO/+)^ mice. **j**, **l** *n*  =  15 cells from five biologically independent *Bdnf*^(tetO/+)^ mice, *n*  =  15 cells from three biologically independent *Iba1*-tTA(+)::*Bdnf*^(tetO/+)^ mice. Data are presented as the mean ± SEM. 2 M: two months of age, control: *Bdnf*^(tetO/+)^ mice, Iba1-Bdnf: *Iba1*-tTA(+)::*Bdnf*^(tetO/+)^ mice.

Given that BDNF is known to be associated with the critical period of experience-dependent neural plasticity [25], we manipulated MG-BDNF overexpression by administering DOX at different time points and measured the social behaviors and electrophysiological properties of the mPFC pyramidal neurons in adulthood to investigate whether MG-BDNF regulates social behavior and mPFC function in a time-specific manner. First, MG-BDNF overexpression during the juvenile period was suppressed by oral DOX administration from p21 in the *Iba1*-tTA::*Bdnf*^(tetO/+)^ mice. Similarly, the control mice were also fed DOX from p21 (Fig. 5a). In the three-chamber social preference test, no difference was detected in the social interaction score or time spent around novel mice between the adult *Iba1*-tTA::*Bdnf*^(tetO/+)^ and control mice at two months of age (Fig. 5b), indicating that the suppressing MG-BDNF overexpression from the juvenile period normalized impaired social behavior in adulthood. No significant differences existed in locomotion or time spent in the center in the open field test (Supplementary Fig. 4). Regarding neuronal function, differences were not observed in spike frequency or amplitude of the excitability of the mPFC layer V pyramidal cells (Fig. 5c, d). Similar results were observed in the sEPSC and mEPSC frequencies (Fig. 5e, f, i, j). In addition, the sIPSC or mIPSC frequency was comparable between the *Iba1*-tTA::*Bdnf*^(tetO/+)^ and control mice (Fig. 5g, h, k, l). These results suggest that normalizing MG-BDNF expression in *Iba1*-tTA::*Bdnf*^(tetO/+)^ from the juvenile period leads to comparable excitability of mPFC layer V pyramidal cells and its inhibitory inputs to the control mice in adulthood.

Next, we assessed the effect of delayed normalization of MG-BDNF overexpression after p45 on social behavior and mPFC layer V pyramidal cell function. DOX was orally administered to the *Iba1*-tTA::*Bdnf*^(tetO/+)^ and control mice from p45–p50 (Supplementary Fig. 5a). The *Iba1*-tTA::*Bdnf*^(tetO/+)^ and control mice exhibited comparable social interaction scores and time spent around the novel mice at two months of age (Supplementary Fig. 5b). Similarly, no differences existed in locomotion or time spent in the center during the open field test (Supplementary Fig. 5c). In contrast, normalizing MG-BDNF during adulthood did not improve the electrophysiological abnormalities of mPFC layer V pyramidal cells in the *Iba1*-tTA::*Bdnf*^(tetO/+)^ mice at two months of age. The spike frequency of excitability remained significantly reduced (Supplementary Fig. 5d). In addition, sIPSC and mIPSC frequencies remained significantly increased in the *Iba1*-tTA::*Bdnf*^(tetO/+)^ mice compared with control mice (Supplementary Fig. 5f, h). Furthermore, the sIPSC amplitude was significantly reduced in the *Iba1*-tTA::*Bdnf*^(tetO/+)^ mice compared with control mice (Supplementary Fig. 5f). No significant differences existed in the frequency or amplitude of sEPSCs or mEPSCs (Supplementary Fig. 5e, g). We also examined whether normalizing the overexpression of MG-BDNF during adulthood could restore complement system-related gene expression changes in the mPFC of *Iba1*-tTA::*Bdnf*^(tetO/+)^ mice. As a result, normalizing MG-BDNF overexpression from p45–p50 restored complement system-related gene expression changes in *Iba1*-tTA::*Bdnf*^(tetO/+)^ mice (Supplementary Fig. 5i).

These results suggest that MG-BDNF overexpression during the juvenile period may be critical for forming inhibitory synapses in the mPFC. However, even the MG-BDNF intervention during adulthood can restore complement system-related gene expression and social behavior.

### *BDNF* expression in human M2 macrophages correlates with childhood experiences

**Fig. 6:**
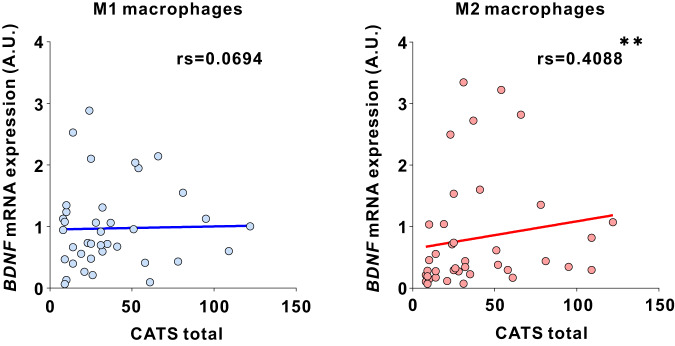
Correlation between human macrophage *BDNF* expression and adverse childhood experiences. (Left) No correlation existed between the total Child Abuse and Trauma Scale (CATS) score and *BDNF* expression in M1 macrophages (rs=0.0694, *p* = 0.6746, Spearman’s rank correlation, *N* = 39). (right) A significant correlation existed between the total CATS score and *BDNF* expression in M2 macrophages (rs=0.4088, *p* = 0.0098, Spearman’s rank correlation, *N* = 39). rs; Spearman’s rank correlation coefficient. The false discovery rate was controlled using the Benjamini–Hochberg method to adjust for multiple comparisons in the CATS total score and Sub-item scores; values with q = 0.033 and *p* < 0.033 were considered significant. **p  <  0.01.

Given the significant correlation in *Bdnf* expression between microglia from the brain and peripheral blood mononuclear cells in GH mice (Supplementary Fig. 6a), we measured *BDNF* expression in human peripheral macrophages, which share properties with microglia [48–50], following the juvenile experience-dependent increase of MG-*Bdnf* in mice. CD14-positive monocytes were collected from the peripheral blood of participants and differentiated into M1/M2 macrophages; subsequently, *BDNF* mRNA expression was measured (Supplementary Fig. 6b). The Japanese version of the Child Abuse Trauma Scale (CATS) was used to assess adverse childhood experiences. The CATS questionnaire consists of 38 self-rated items that retrospectively assess the frequency of adverse event experiences in childhood and adolescence in five categories: sexual abuse, physical abuse, emotional abuse, neglect, and others, with participants rating each item on a scale of 0 to 4 and total CATS score reflects severity [51, 52]. A positive correlation existed between the total CATS scores and *BDNF* expression in M2 macrophages (Fig. 6). In the CATS sub-items, neglect and punishment, among other variables, were positively correlated with *BDNF* expression in M2 macrophages (Supplementary Table 1). In contrast, no significant correlations existed between *BDNF* expression in M1 macrophages and the CATS total or sub-item scores (Fig. 6 and Supplementary Table 1). These results indicate that adverse childhood experiences may increase *BDNF* expression in M2 macrophages, even in humans.

## Discussion

Little is known concerning the microglia’s influence on mPFC development and its function, such as social behavior [53]. As such, microglia are receiving significantly more attention in psychiatric research [54, 55]. In particular, understanding microglia’s role in the mPFC circuit formation is essential due to the critical implication of the mPFC in the pathobiology of neuropsychiatric disorders [53, 56–58]. Our study identified the critical role of microglial BDNF in social behaviors, cellular excitability, and synaptic functions in the mPFC. BDNF overexpression limited to microglia was sufficient to recapitulate social behavior deficits and reduce the excitability of layer V pyramidal cells in the mPFC. To critically evaluate the necessity of MG-BDNF overexpression, we normalized it from p21 or p45. Both treatments normalized social behavior deficits, but their impacts on the excitability of layer V pyramidal cells differed. The normalization of MG-BDNF overexpression from p21 restored the spike frequency, amplitude, and frequencies of sEPSC, mEPSC, sIPSC, and mIPSC. By contrast, the normalization of MG-BDNF overexpression starting at p45 restored the spike amplitude and frequencies of sEPSC and mEPSC but failed to normalize a reduced spike frequency and increased frequencies of sIPSC and mIPSC. While excitatory tones and social behaviors changed in the same direction in response to MG-BDNF normalization, such correlated changes were not seen between social behaviors and inhibitory synaptic functions. A parsimonious interpretation of this set of observations is that social behavior deficits caused by MG-BDNF are primarily mediated by the reduced excitatory tone in the mPFC. Our data also showed intriguing dissociations between social behavior and electrophysiological phenotypes. Despite normalized social behaviors, the spike frequency remained depressed, and sIPSC and mIPSC frequencies remained elevated following MG-BDNF normalization starting at p45. These data, thus, do not support the functional relevance of these cellular excitability and synaptic functions to social behaviors. Moreover, our data do not lend strong support for the role of the excitatory/inhibitory balance in social behaviors. A shift toward a more inhibitory tone was seen in the presence of constant MG-BDNF overexpression (see Fig. 3d–k; reduced sEPSC and mEPSC and increased sIPSC and mIPSC) and MG-BDNF normalization from p45 (see Supplementary Fig. 5e-h; normalized sEPSC and mEPSC, and increased sIPSC and mIPSC) despite the fact social behaviors were reduced and normalized, respectively. More work is required to critically evaluate the causal role of the excitatory/inhibitory balance in social behavior. Our observations provide a starting point to delve into causative links—or lack thereof—among MG-BDNF and cellular excitability and synaptic functions as molecular and cellular substrates of social behaviors.

Recently, Schalbetter et al. reported that microglia affect mPFC function, including cognitive function, in a time-specific manner [24]. Although microglia are reportedly related to social behavior [20–22], no study has examined the relationship between sociability and time-specific development of the mPFC with microglia. The j-SI mouse with robust impairment of social behavior is a potential model for human neglect [11, 12]; however, it also makes it possible to elucidate the mechanism of social circuit formation in a limited social experience-dependent window, similar to that of sensory deprivation [59]. Social experience deprivation during the juvenile period (p21–35) has been suggested to affect the excitatory/inhibitory balance in mPFC [16–18], mPFC–pPVT neural circuits [14], and glial cells, such as oligodendrocytes [12] and microglia [13], all of which may be responsible for reduced sociability in these mice. Following the current finding that juvenile social experience deprivation elevates MG-BDNF expression in mPFC, a novel mechanism could elucidate the experience-dependent development of social behaviors. In mice overexpressing MG-BDNF, we demonstrated that higher MG-BDNF expression reduced social behaviors, suggesting that MG-BDNF may be critical in developing social abilities. In addition to a robust social assessment, i.e., the three-chamber social test, we also applied the AR-LABO, in which multiple mice were simultaneously traced under free-moving conditions to confirm their social behavior. MG-BDNF-overexpressing mice exhibited fewer approaches to other mice and received fewer approaches from others. This might be due to MG-BDNF-overexpressing mice emitting reduced levels of communication through ultrasonic vocalizations and lower levels of chemical communication, such as urine and pheromones [60, 61].

Furthermore, MG-BDNF modulates the excitability and excitatory/inhibitory input of mPFC layer V pyramidal cells in a limited window from the juvenile period (p21) to adulthood (p45–p50). This is consistent with the critical period for social ability acquisition in mice, which is from p21 to p35 [12]. In contrast, normalizing MG-BDNF during adulthood did not improve the excitability and excitatory/inhibitory balance of mPFC layer V pyramidal cells, although impaired social behavior was ameliorated. MG-BDNF is implicated in learning-dependent neural plasticity [29] and may modify social circuit formation in brain regions other than the mPFC, even in adulthood [62]. For example, the high-frequency deep brain stimulation in the anterior insula, which is a well-known brain region regulating social behavior, improves the impairment of sociability and social novelty preference, but not spatial learning abilities and cognitive rigidity, in valproic acid-exposed rats [63]. Previous studies have also reported that microglia are related to mPFC circuit formation and cognitive maturation in adolescence [24]; thus, normalizing MG-BDNF after p45–p50 may be sufficient to restore sociability. Understanding these mechanisms and interactions is a future research challenge for further investigation.

The excitability of layer V pyramidal cells in the mPFC of MG-BDNF-overexpressing mice is similar to that observed in j-SI mice [16, 18]. This reduction in the excitability of layer V mPFC neurons may be associated with the hypoactivity of mPFC neurons that project subcortically to regulate social behavior [14], leading to reduced sociability. The relationship between MG-BDNF and the development and maturation of inhibitory neuronal circuits is poorly understood; however, enhancing inhibitory neuronal circuits, as in this study, is likely consistent with a known function of BDNF: promoting the formation and maintenance of inhibitory neural synapses during brain development [25, 64–66]. Particularly, BDNF regulates the critical visual cortex period, and overexpressed BDNF leads to the premature maturation of inhibitory neural circuits, leading to early closure of the critical visual cortex period [25]. Juvenile PFC development strengthens inhibitory neurotransmission within the brain, altering the excitatory/inhibitory balance [67, 68], which is implicated in the social function of the mPFC [19]. Overexpressed MG-BDNF might similarly close the critical social development window, disrupting mPFC development and reducing sociability by strengthening inhibitory neural circuits. In this study, normalizing MG-BDNF from adulthood (p45–p50) did not ameliorate the enhanced inhibitory neuronal circuitry. In the rodent neocortex, inhibitory synapse formation primarily occurs postnatally and rapidly (before adolescence) reaches adult-like inhibitory synapse density [69, 70]. The time course of rodent inhibitory synapse functional maturation is similar to that of inhibitory synapse formation. Specifically, IPSC frequency becomes prominent postnatally and displays adult-like properties before adolescence [71]. Enhancing inhibitory neuronal circuits via overexpressed MG-BDNF may increase the density and function of inhibitory synapses. Previous studies have also revealed increased inhibitory inputs in mPFC layer V pyramidal cells in j-SI mice and other abnormalities in inhibitory interneuron functions in the mPFC [11, 15, 17, 18]. Abnormalities in inhibitory circuits induced by juvenile isolation and changes in MG-BDNF expression might be a potential mechanism for the experience-dependent impairment of social development.

In this study, we performed RNA-seq of the mPFC; our findings suggested the involvement of the complement system as a mechanism of MG-BDNF-induced reduction of sociability. The relationship between BDNF and the complement system has not previously been reported; nevertheless, complement C3 signaling starting at C1q is crucial for the experience-dependent synaptic pruning of microglia [2, 7]. Thus, decreased *C1q* and *C3ar1* [72–75] expression may reduce microglial pruning of both excitatory and inhibitory synapses [76], and the subsequent excitatory/inhibitory imbalance may cause the current findings in this study. In this study, normalization of the complement system from adulthood did not restore the mPFC circuit abnormalities, suggesting that complement-mediated maturation of the mPFC may particularly occur in juveniles. Recent research has suggested that microglia are the primary source of *C1q* [77]; however, neurons also express *C1q* [78]. In this study, although we could not definitively establish whether the alterations in the complement system across the entire mPFC are solely attributed to microglia, the fact that MG-BDNF overexpression did not independently induce substantial changes in the expression of complement system-related genes within cortical microglia suggests the possibility that these changes are specific to microglia in the mPFC or could be the result of complement interactions with neurons and other glial cells [79]. In addition, the relationship between the complement system and psychiatric disorders is gradually becoming more evident [80, 81], indicating that further investigations are needed.

Our experiments with mice have several limitations. First, we used *Bdnf* ^(tetO/+)^ mice as the control group and *Iba1*-tTA::*Bdnf* ^(tetO/+)^ mice as the experimental group. Our *Bdnf* ^(tetO/+)^ mice were derived from ES cells of 129/SvEv mice for homologous recombination and backcrossed with C57BL/6 J mice for more than five generations. While *Iba1*-tTA mice were originally developed in fertilized eggs of C57BL/6 J, they were maintained as breeders with our *Bdnf* ^(tetO/+)^ mice. Accordingly, the alleles near the transgene *Iba1*-tTA are expected to be enriched with those of C57BL/6 J and alleles in the rest of the genome contained randomly mixed 129/SvEv and C57BL/6 J alleles originating from *Bdnf* ^(tetO/+)^ mice. Thus, the expected impacts of a systematic genetic background bias between the control and experimental groups were minimized, as the alleles near *Iba1*-tTA transgene are those of C57BL/6 J in both control and experimental groups and the rest of the genome contained a random mixture of 129/SvEv and C57BL/6 J alleles. The mixed genetic backgrounds of *Bdnf* ^(tetO/+)^ mice were present in both the control and experimental groups [34]. Moreover, if the phenotypes reflected genetic background differences between control and experimental groups instead of or in addition to MG-BDNF overexpression, some phenotypic differences between the two groups should have remained after normalizing MG-BDNF levels; no phenotypic difference was seen in social behavior (Fig. 5b) or electrophysiological recordings (Fig. 5c–l). These results suggest that the genetic mixed background is minimal. Second, the duration during which MG-BDNF overexpression remained suppressed may be critical rather than the timing of the DOX administration initiation. However, we have shown that resocialization after p35 in j-SI mice does not improve either sociability or mPFC function [12, 16–18], which may support the timing specificity in the current study. Third, similar to microglia, astrocytes are involved in synaptic plasticity through BDNF [82–84]. Precursor pro-BDNF proteins, secreted by neurons, are captured by astrocytes via endocytosis, after which pro-BDNFs are converted into mature BDNFs [85]. Astrocytes also play a role in BDNF storage and may regulate BDNF levels in the brain [86]. Although no previous studies have demonstrated direct involvement of astrocytic BDNF in social behavior, astrocytic BDNF has been implicated in memory [84], and the overexpression of astrocytic BDNF induces anxiolytic/antidepressant-like behavior in mice [87]. These facts suggest that astrocytic BDNF may also affect social behavior. Furthermore, while our study was primarily centered on *Bdnf* expression in microglia within the mPFC, the status of *Bdnf* in microglia in other brain regions remains to be discovered. Additionally, the impact of MG-BDNF overexpression on the electrophysiological functions of brain regions has only been elucidated in the mPFC and pPVT. Further research is required to investigate these challenges.

Childhood experiences were also associated with *BDNF* expression in human peripheral M2 macrophages in this study. Microglia and macrophages should be considered separately [48], as primitive myeloid progenitor cells (microglia’s origin) migrate from the yolk sac into the brain from the embryonic period [88]. It is possible that we only observe common phenotypes in microglia and macrophages induced by certain upstream molecules [89]. Additionally, we assessed the relationship between *Bdnf* mRNA expression in microglia and the periphery in mice peripheral blood mononuclear cells, not in macrophages. Furthermore, it is unclear whether j-SI in mice increases *Bdnf* mRNA levels in macrophages. Thus, results in mice and humans cannot be directly compared; however, a relationship exists between childhood experiences and *BDNF* expression in macrophages that share similarities in CD11b expression and phagocytic capacity with microglia [48, 49] (the resident macrophages in the brain [49, 50]). M1 macrophages have high antigen-presenting activity and pro-inflammatory cytokine-releasing capacity. In contrast, M2 macrophages have multiple roles aside from inflammation, including anti-inflammatory responses and tissue remodeling, and secrete numerous growth and neurotrophic factors [90–92]. M2 macrophages are also implicated in the pathobiology of neuropsychiatric and neurodegenerative disorders [93–95]. Whether high levels of *BDNF* in M2 macrophages are associated with reduced sociability remains unclear; however, BDNF abnormalities have been identified in humans with autism spectrum and posttraumatic stress disorders [96–98]. They may also be associated with the reduced sociability of these disorders [99, 100]. The CATS used for assessing childhood abuse and trauma in this study is a self-rated questionnaire. Notably, self-rated assessments could be inherently more variable compared to more objective measurements obtained by trained clinical staff.

In conclusion, these findings indicate that MG-BDNF is critical in developing social behaviors and mPFC function in a time-specific manner, potentially related to juvenile social experience-dependent social development. Our results provide new insights into experience-dependent social behavior formation and mPFC development.

### Supplementary information

sFigure 1

sFigure 2

sFigure 3

sFigure 4

sFigure 5

sFigure 6

sTable 1

Supplemental Manuscript

## Data Availability

The datasets generated and/or analyzed during the current study are available from the corresponding author on reasonable request.
