# Supplementary material for: Brain-derived neurotrophic factor from microglia regulates neuronal development in the medial prefrontal cortex and its associated social behavior
Source: Mol Psychiatry. Author manuscript; Available in PMC 2024 Jun 21. (PMC11189755; doi:10.1038/s41380-024-02413-y)
Supplement: Suppl. Figures and Table [file NIHMS1971768-supplement-Suppl__Figures_and_Table.pdf]

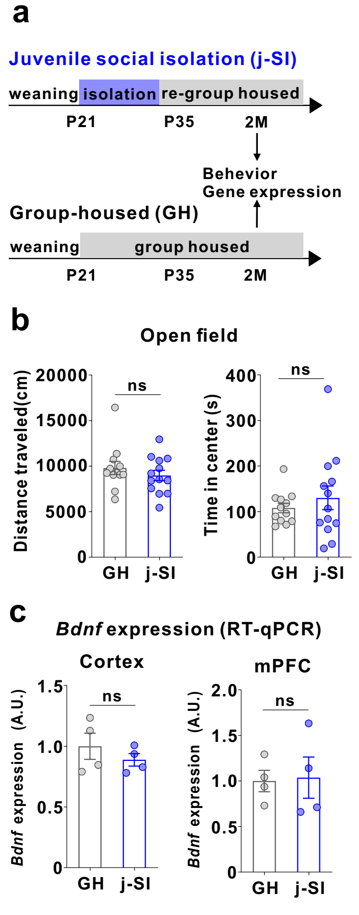

a
Juvenile social isolation (j-SI)
weaning
isolation
re-group housed
P21
P35
2M
Behavior
Gene expression
Group-housed (GH)
weaning
group housed
P21
P35
2M
b
Open field
Distance traveled(cm)
20000
15000
10000
5000
0
ns
GH
j-SI
Time in center (s)
400
300
200
100
0
ns
GH
j-SI
c
Bdnf expression (RT-qPCR)
Cortex
Bdnf expression (A.U.)
1.5
1.0
0.5
0
ns
GH
j-SI
mPFC
Bdnf expression (A.U.)
2.0
1.5
1.0
0.5
0
ns
GH
j-SI

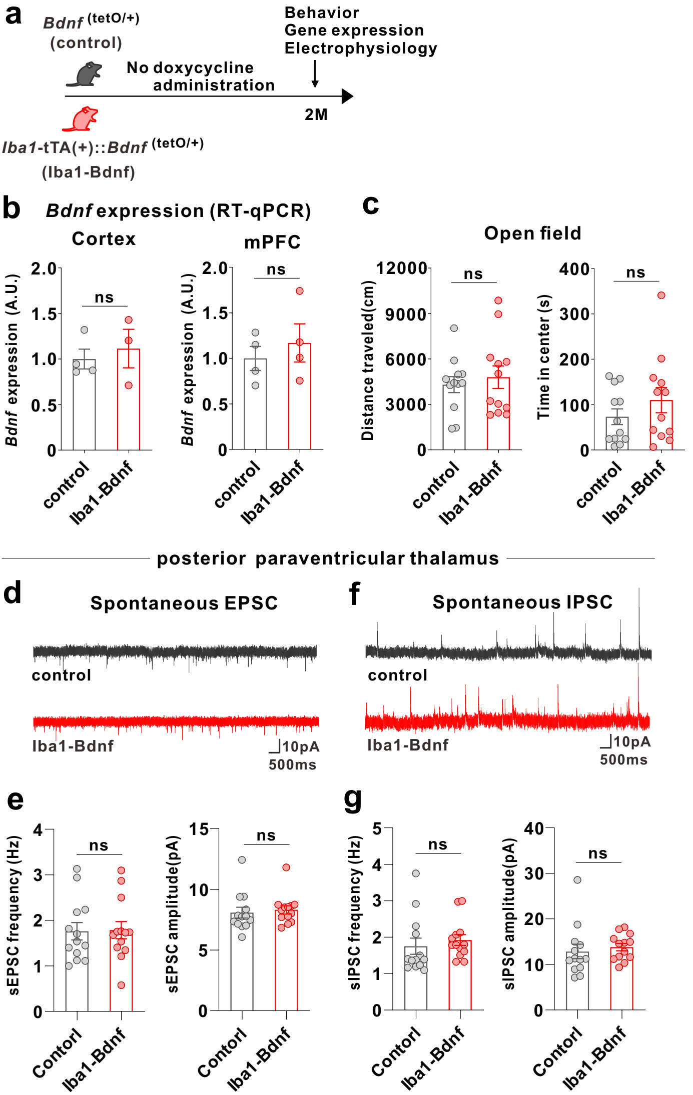

a
Bdnf (tetO/+)
(control)
No doxycycline administration
Behavior
Gene expression
Electrophysiology
2M
Iba1-tTA(+)::Bdnf (tetO/+)
(Iba1-Bdnf)
b
Bdnf expression (RT-qPCR)
Cortex
mPFC
Bdnf expression (A.U.)
ns
control
Iba1-Bdnf
c
Open field
Distance traveled(cm)
Time in center (s)
ns
control
Iba1-Bdnf
posterior paraventricular thalamus
d
Spontaneous EPSC
control
Iba1-Bdnf
10pA
500ms
f
Spontaneous IPSC
control
Iba1-Bdnf
10pA
500ms
e
sEPSC frequency (Hz)
sEPSC amplitude(pA)
ns
Contorl
Iba1-Bdnf
g
sIPSC frequency (Hz)
sIPSC amplitude(pA)
ns
Contorl
Iba1-Bdnf

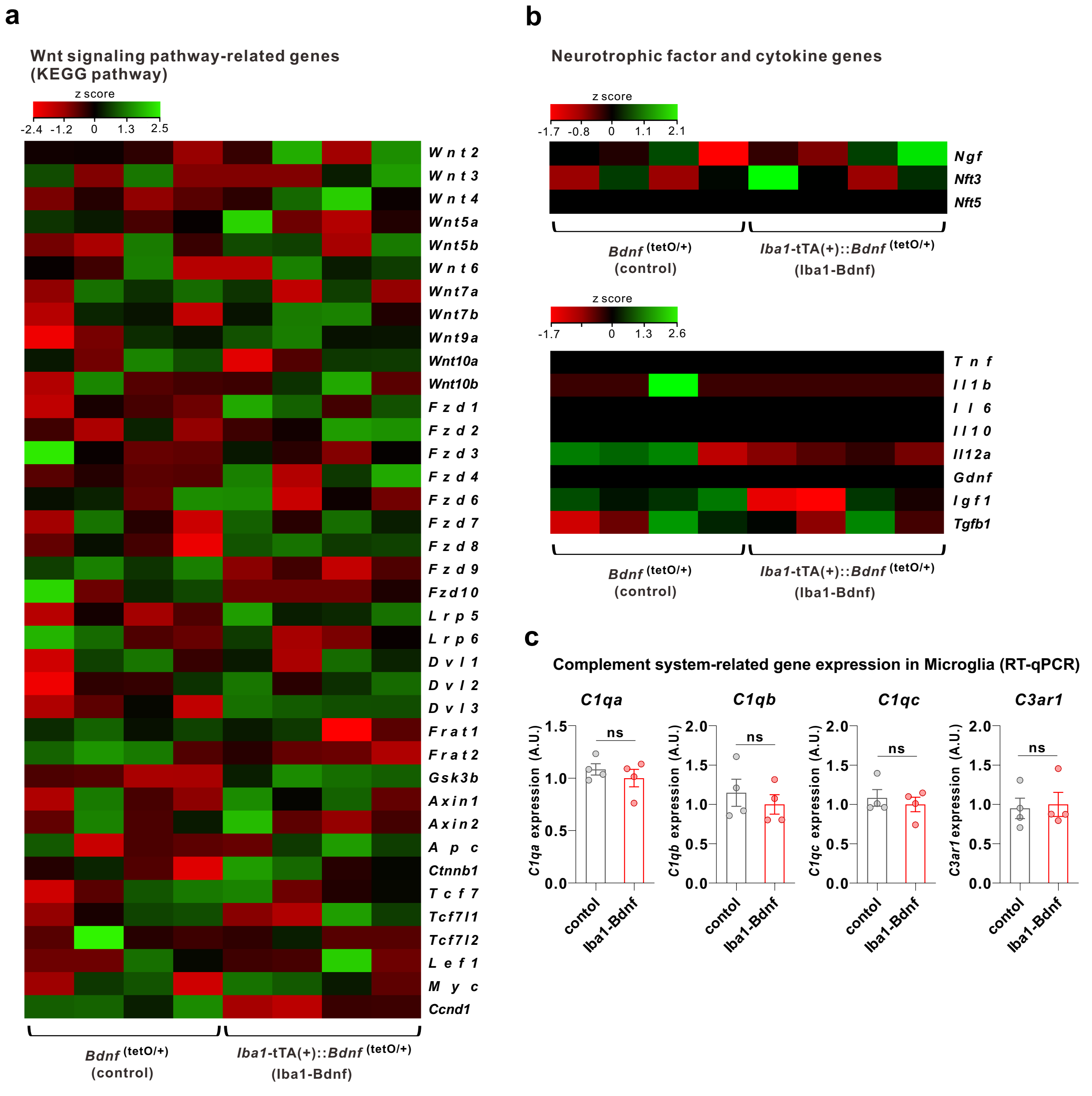
a
Wnt signaling pathway-related genes (KEGG pathway)
z score
-2.4 -1.2 0 1.3 2.5
Wnt2
Wnt3
Wnt4
Wnt5a
Wnt5b
Wnt6
Wnt7a
Wnt7b
Wnt9a
Wnt10a
Wnt10b
Fzd1
Fzd2
Fzd3
Fzd4
Fzd6
Fzd7
Fzd8
Fzd9
Fzd10
Lrp5
Lrp6
Dvl1
Dvl2
Dvl3
Frat1
Frat2
Gsk3b
Axin1
Axin2
Apc
Ctnnb1
Tcf7
Tcf7l1
Tcf7l2
Lef1
Myc
Ccnd1
Bdnf (tetO/+) (control)
Iba1-tTA(+)::Bdnf (tetO/+) (Iba1-Bdnf)
b
Neurotrophic factor and cytokine genes
z score
-1.7 -0.8 0 1.1 2.1
Ngf
Nft3
Nft5
Bdnf (tetO/+) (control)
Iba1-tTA(+)::Bdnf (tetO/+) (Iba1-Bdnf)
z score
-1.7 0 1.3 2.6
Tnf
Il1b
Il6
Il10
Il12a
Gdnf
Igf1
Tgfb1
Bdnf (tetO/+) (control)
Iba1-tTA(+)::Bdnf (tetO/+) (Iba1-Bdnf)
c
Complement system-related gene expression in Microglia (RT-qPCR)
C1qa
C1qb
C1qc
C3ar1
C1qa expression (A.U.)
C1qb expression (A.U.)
C1qc expression (A.U.)
C3ar1 expression (A.U.)
ns
contol
Iba1-Bdnf
0.0 0.5 1.0 1.5 2.0

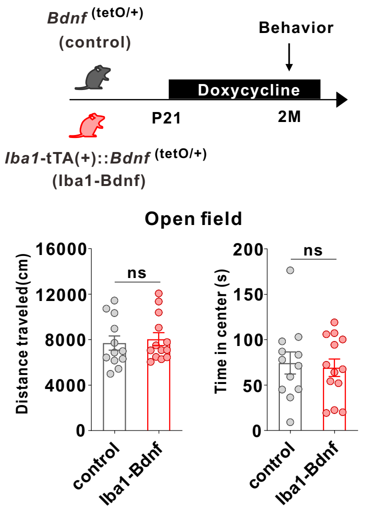

Bdnf (tetO/+)
(control)
Behavior
Doxycycline
P21
2M
Iba1-tTA(+)::Bdnf (tetO/+)
(Iba1-Bdnf)
Open field
Distance traveled(cm)
16000
12000
8000
4000
0
ns
control
Iba1-Bdnf
Time in center (s)
200
150
100
50
0
ns
control
Iba1-Bdnf

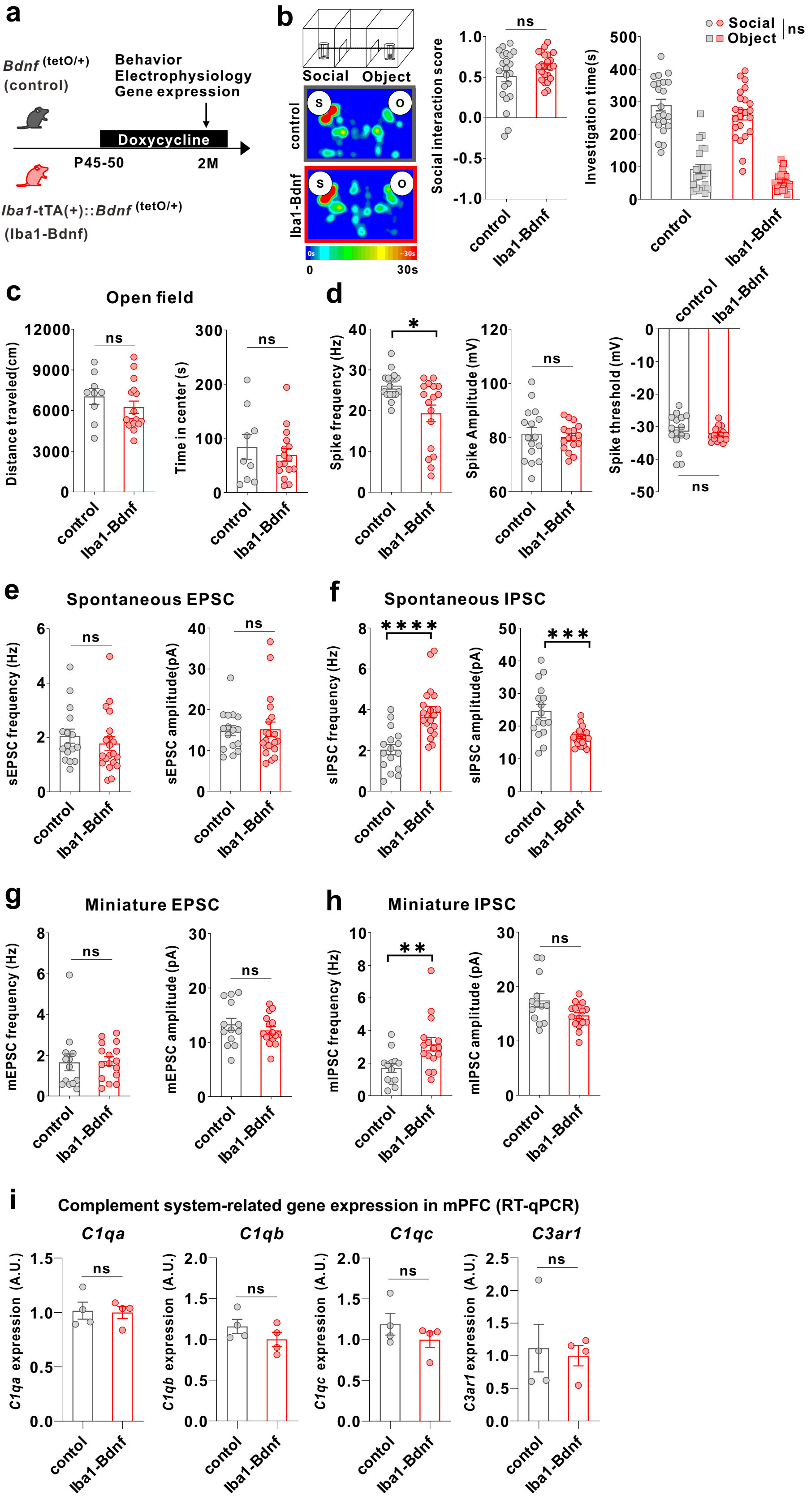

a
Bdnf (tetO/+) (control)
Behavior
Electrophysiology
Gene expression
Doxycycline
P45-50
2M
Iba1-tTA(+)::Bdnf (tetO/+) (Iba1-Bdnf)
b
Social
Object
control
Iba1-Bdnf
0s
30s
Social interaction score
Investigation time(s)
Social
Object
ns
c
Open field
Distance traveled(cm)
Time in center (s)
d
Spike frequency (Hz)
Spike Amplitude (mV)
Spike threshold (mV)
e
Spontaneous EPSC
sEPSC frequency (Hz)
sEPSC amplitude(pA)
f
Spontaneous IPSC
sIPSC frequency (Hz)
sIPSC amplitude(pA)
g
Miniature EPSC
mEPSC frequency (Hz)
mEPSC amplitude (pA)
h
Miniature IPSC
mIPSC frequency (Hz)
mIPSC amplitude (pA)
i
Complement system-related gene expression in mPFC (RT-qPCR)
C1qa
C1qb
C1qc
C3ar1
C1qa expression (A.U.)
C1qb expression (A.U.)
C1qc expression (A.U.)
C3ar1 expression (A.U.)
contol
Iba1-Bdnf

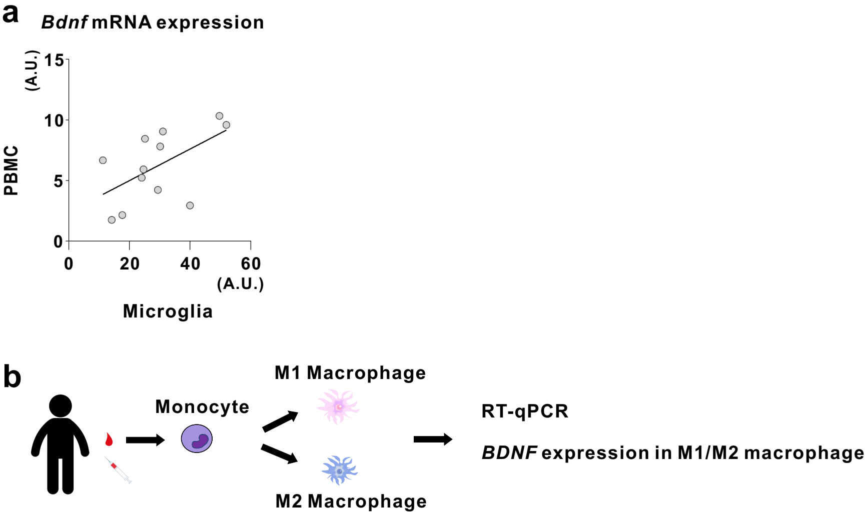

a
Bdnf mRNA expression
PBMC (A.U.)
15
10
5
0
0
20
40
60
(A.U.)
Microglia
b
Monocyte
M1 Macrophage
M2 Macrophage
RT-qPCR
BDNF expression in M1/M2 macrophage

Supplementary Table 1. *BDNF* expression in Macrophages

	M1		M2	
CATS	rs	p-value	rs	p-value
Sub-item scores				
Sexual abuse	-0.114	0.488	0.293	0.071
Punishment	0.230	0.159	0.396	0.013 *
Neglect	0.008	0.960	0.348	0.030 *
Emotional abuse	-0.043	0.795	0.321	0.046
Others	0.093	0.574	0.367	0.022 *
