# Supplementary material for: Brain-derived neurotrophic factor from microglia regulates neuronal development in the medial prefrontal cortex and its associated social behavior
Source: Mol Psychiatry. 2024 Jan 19;29(5):1338–49. doi: 10.1038/s41380-024-02413-y (PMC11189755; doi:10.1038/s41380-024-02413-y)
Supplement: Supplementary file 1 [file 41380_2024_2413_MOESM1_ESM.pdf]

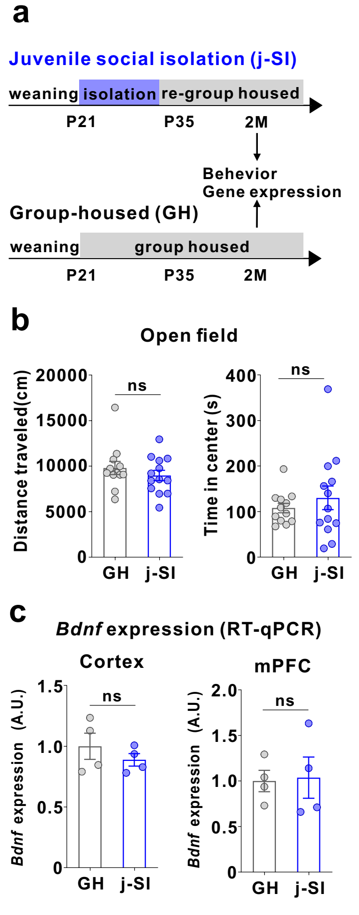
a
Juvenile social isolation (j-SI)
weaning
isolation
re-group housed
P21
P35
2M
Behavior
Gene expression
Group-housed (GH)
weaning
group housed
P21
P35
2M
b
Open field
Distance traveled(cm)
20000
15000
10000
5000
0
ns
GH
j-SI
Time in center (s)
400
300
200
100
0
ns
GH
j-SI
c
Bdnf expression (RT-qPCR)
Cortex
Bdnf expression (A.U.)
1.5
1.0
0.5
0
ns
GH
j-SI
mPFC
Bdnf expression (A.U.)
2.0
1.5
1.0
0.5
0
ns
GH
j-SI
