# Supplementary material for: Brain-derived neurotrophic factor from microglia regulates neuronal development in the medial prefrontal cortex and its associated social behavior
Source: Mol Psychiatry. 2024 Jan 19;29(5):1338–49. doi: 10.1038/s41380-024-02413-y (PMC11189755; doi:10.1038/s41380-024-02413-y)
Supplement: Supplementary file 2 [file 41380_2024_2413_MOESM2_ESM.pdf]

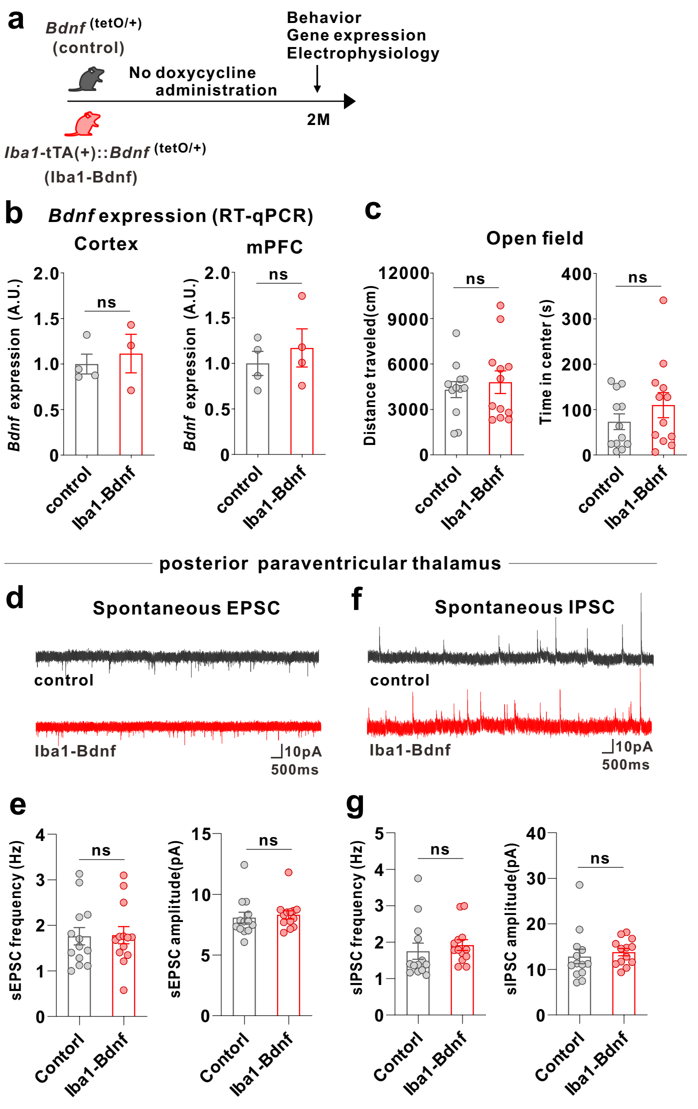
a
Bdnf (tetO/+)
(control)
No doxycycline administration
Behavior
Gene expression
Electrophysiology
2M
Iba1-tTA(+)::Bdnf (tetO/+)
(Iba1-Bdnf)
b
Bdnf expression (RT-qPCR)
Cortex
mPFC
Bdnf expression (A.U.)
ns
control
Iba1-Bdnf
c
Open field
Distance traveled(cm)
Time in center (s)
posterior paraventricular thalamus
d
Spontaneous EPSC
control
Iba1-Bdnf
10pA
500ms
f
Spontaneous IPSC
e
sEPSC frequency (Hz)
sEPSC amplitude(pA)
Contorl
g
sIPSC frequency (Hz)
sIPSC amplitude(pA)
