# Supplementary material for: Brain-derived neurotrophic factor from microglia regulates neuronal development in the medial prefrontal cortex and its associated social behavior
Source: Mol Psychiatry. 2024 Jan 19;29(5):1338–49. doi: 10.1038/s41380-024-02413-y (PMC11189755; doi:10.1038/s41380-024-02413-y)
Supplement: Supplementary file 3 [file 41380_2024_2413_MOESM3_ESM.pdf]

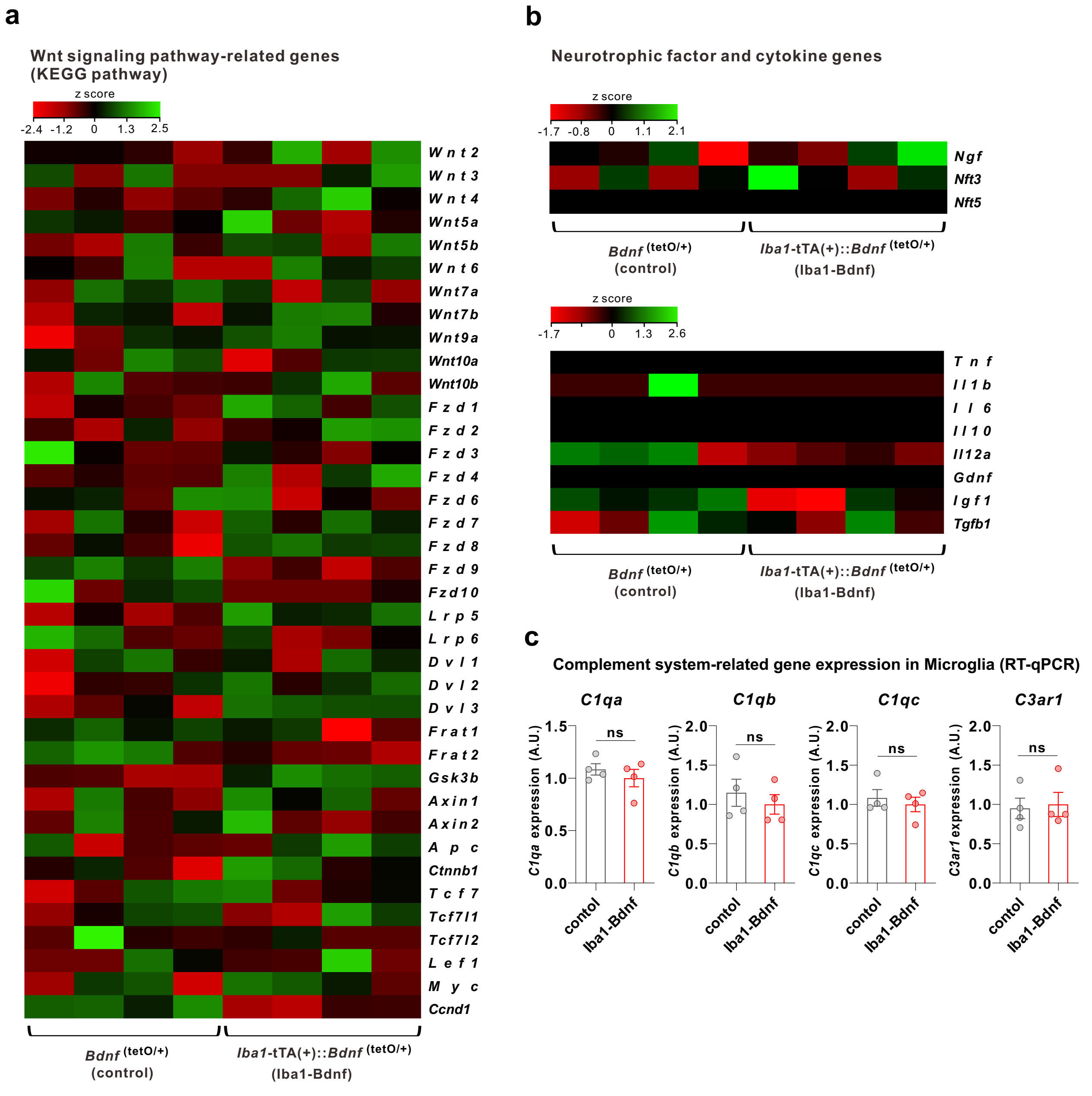
a
Wnt signaling pathway-related genes (KEGG pathway)
z score
-2.4 -1.2 0 1.3 2.5
Wnt2
Wnt3
Wnt4
Wnt5a
Wnt5b
Wnt6
Wnt7a
Wnt7b
Wnt9a
Wnt10a
Wnt10b
Fzd1
Fzd2
Fzd3
Fzd4
Fzd6
Fzd7
Fzd8
Fzd9
Fzd10
Lrp5
Lrp6
Dvl1
Dvl2
Dvl3
Frat1
Frat2
Gsk3b
Axin1
Axin2
Apc
Ctnnb1
Tcf7
Tcf7l1
Tcf7l2
Lef1
Myc
Ccnd1
Bdnf (tetO/+) (control)
Iba1-tTA(+)::Bdnf (tetO/+) (Iba1-Bdnf)
b
Neurotrophic factor and cytokine genes
z score
-1.7 -0.8 0 1.1 2.1
Ngf
Nft3
Nft5
Bdnf (tetO/+) (control)
Iba1-tTA(+)::Bdnf (tetO/+) (Iba1-Bdnf)
z score
-1.7 0 1.3 2.6
Tnf
Il1b
Il6
Il10
Il12a
Gdnf
Igf1
Tgfb1
Bdnf (tetO/+) (control)
Iba1-tTA(+)::Bdnf (tetO/+) (Iba1-Bdnf)
c
Complement system-related gene expression in Microglia (RT-qPCR)
C1qa
C1qa expression (A.U.)
1.5
1.0
0.5
0.0
ns
contol
Iba1-Bdnf
C1qb
C1qb expression (A.U.)
2.0
1.5
1.0
0.5
0.0
ns
contol
Iba1-Bdnf
C1qc
C1qc expression (A.U.)
2.0
1.5
1.0
0.5
0.0
ns
contol
Iba1-Bdnf
C3ar1
C3ar1 expression (A.U.)
2.0
1.5
1.0
0.5
0.0
ns
contol
Iba1-Bdnf
