# Supplementary material for: Brain-derived neurotrophic factor from microglia regulates neuronal development in the medial prefrontal cortex and its associated social behavior
Source: Mol Psychiatry. 2024 Jan 19;29(5):1338–49. doi: 10.1038/s41380-024-02413-y (PMC11189755; doi:10.1038/s41380-024-02413-y)
Supplement: Supplementary file 4 [file 41380_2024_2413_MOESM4_ESM.pdf]

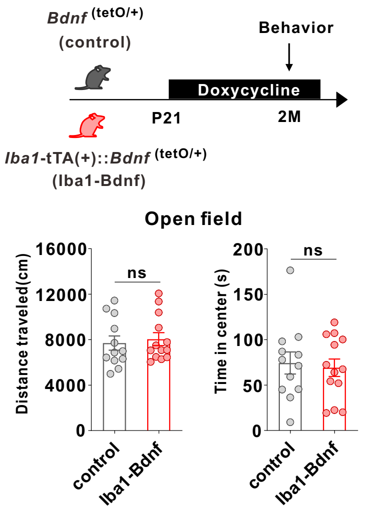

Bdnf (tetO/+)
(control)
Behavior
Doxycycline
P21
2M
Iba1-tTA(+)::Bdnf (tetO/+)
(Iba1-Bdnf)
Open field
Distance traveled(cm)
16000
12000
8000
4000
0
ns
control
Iba1-Bdnf
Time in center (s)
200
150
100
50
0
ns
control
Iba1-Bdnf
