# Supplementary material for: Brain-derived neurotrophic factor from microglia regulates neuronal development in the medial prefrontal cortex and its associated social behavior
Source: Mol Psychiatry. 2024 Jan 19;29(5):1338–49. doi: 10.1038/s41380-024-02413-y (PMC11189755; doi:10.1038/s41380-024-02413-y)
Supplement: Supplementary file 5 [file 41380_2024_2413_MOESM5_ESM.pdf]

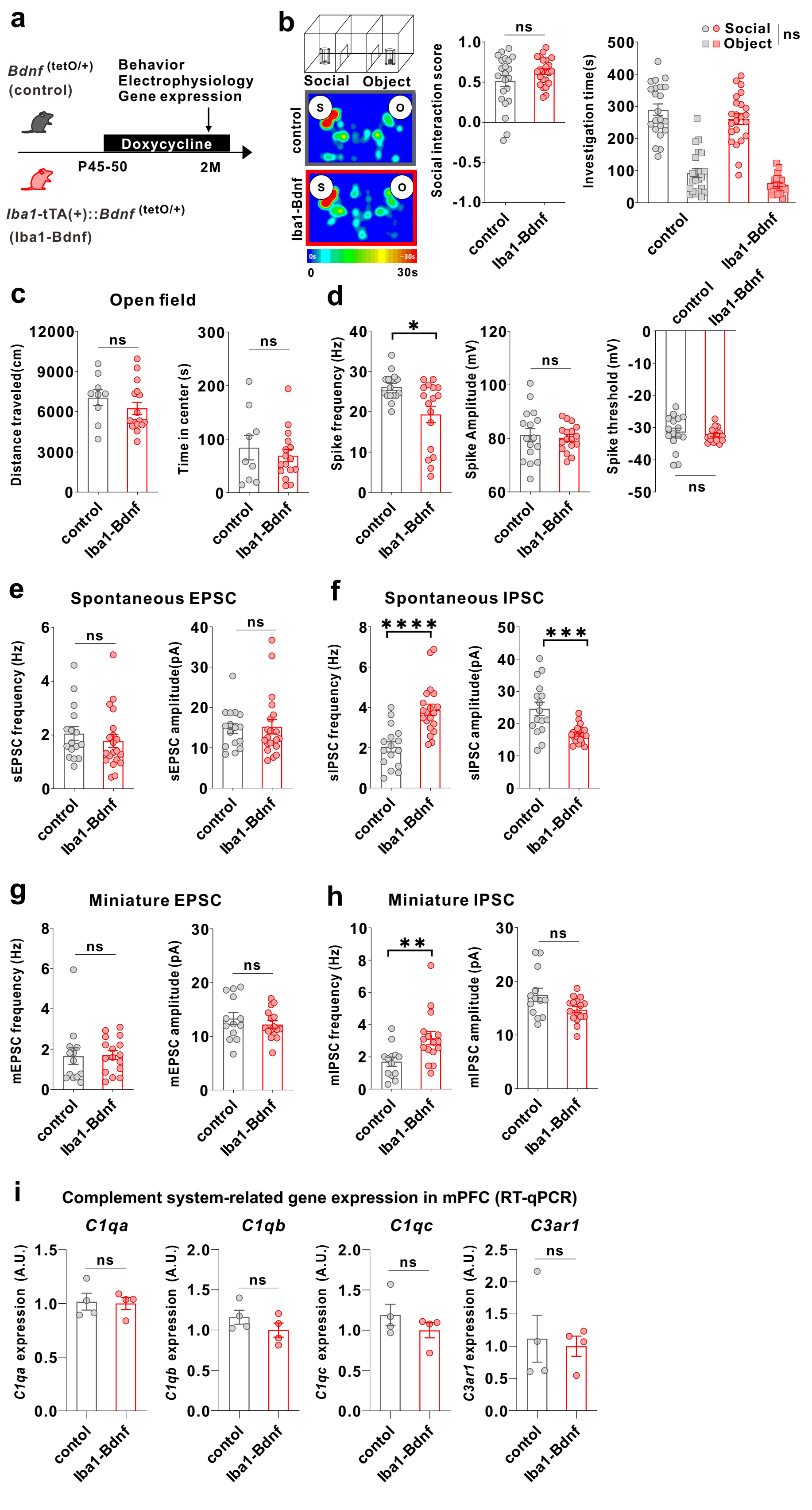

a
Bdnf (tetO/+)
(control)
Behavior
Electrophysiology
Gene expression
Doxycycline
P45-50
2M
Iba1-tTA(+)::Bdnf (tetO/+)
(Iba1-Bdnf)
b
Social
Object
control
Iba1-Bdnf
S
O
0
30s
Social interaction score
Investigation time(s)
Social
Object
ns
c
Open field
Distance traveled(cm)
Time in center (s)
d
Spike frequency (Hz)
Spike Amplitude (mV)
Spike threshold (mV)
e
Spontaneous EPSC
sEPSC frequency (Hz)
sEPSC amplitude(pA)
f
Spontaneous IPSC
sIPSC frequency (Hz)
sIPSC amplitude(pA)
g
Miniature EPSC
mEPSC frequency (Hz)
mEPSC amplitude (pA)
h
Miniature IPSC
mIPSC frequency (Hz)
mIPSC amplitude (pA)
i
Complement system-related gene expression in mPFC (RT-qPCR)
C1qa
C1qb
C1qc
C3ar1
C1qa expression (A.U.)
C1qb expression (A.U.)
C1qc expression (A.U.)
C3ar1 expression (A.U.)
control
contol
Iba1-Bdnf
