# Supplementary material for: Brain-derived neurotrophic factor from microglia regulates neuronal development in the medial prefrontal cortex and its associated social behavior
Source: Mol Psychiatry. 2024 Jan 19;29(5):1338–49. doi: 10.1038/s41380-024-02413-y (PMC11189755; doi:10.1038/s41380-024-02413-y)
Supplement: Supplementary file 6 [file 41380_2024_2413_MOESM6_ESM.pdf]

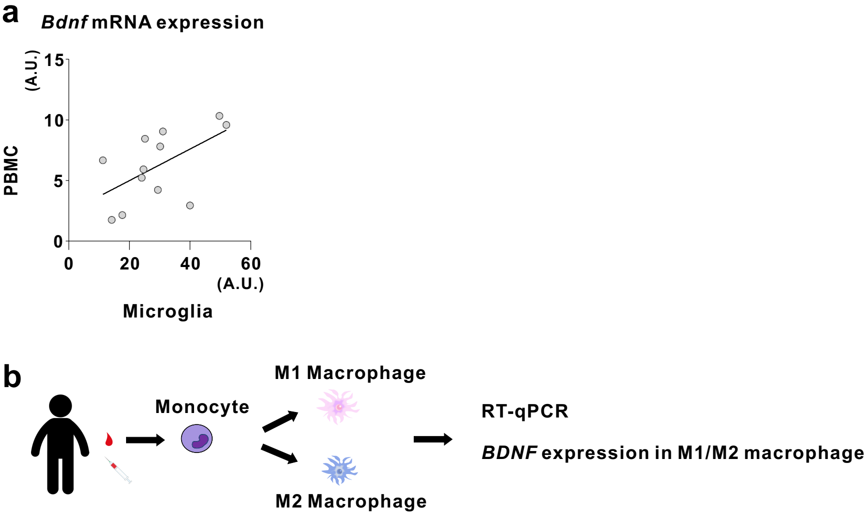

a
Bdnf mRNA expression
PBMC
(A.U.)
15
10
5
0
0
20
40
60
(A.U.)
Microglia
b
Monocyte
M1 Macrophage
M2 Macrophage
RT-qPCR
BDNF expression in M1/M2 macrophage
