# Supplementary material for: Brain-derived neurotrophic factor from microglia regulates neuronal development in the medial prefrontal cortex and its associated social behavior
Source: Mol Psychiatry. 2024 Jan 19;29(5):1338–49. doi: 10.1038/s41380-024-02413-y (PMC11189755; doi:10.1038/s41380-024-02413-y)
Supplement: Supplementary file 7 [file 41380_2024_2413_MOESM7_ESM.docx]

Supplementary Table 1. *BDNF* expression in Macrophages

|  | M1 |  |  | M2 |  |
| --- | --- | --- | --- | --- | --- |
| CATS | rs | p-value |  | rs | p-value |
| Sub-item scores |  |  |  |  |  |
| Sexual abuse | -0.114 | 0.488 |  | 0.293 | 0.071 |
| Punishment | 0.230 | 0.159 |  | 0.396 | 0.013 ***** |
| Neglect | 0.008 | 0.960 |  | 0.348 | 0.030 ***** |
| Emotional abuse | -0.043 | 0.795 |  | 0.321 | 0.046 |
| Others | 0.093 | 0.574 |  | 0.367 | 0.022 ***** |
