# Supplementary material for: Brain-derived neurotrophic factor from microglia regulates neuronal development in the medial prefrontal cortex and its associated social behavior
Source: Mol Psychiatry. 2024 Jan 19;29(5):1338–49. doi: 10.1038/s41380-024-02413-y (PMC11189755; doi:10.1038/s41380-024-02413-y)
Supplement: Supplementary file 8 [file 41380_2024_2413_MOESM8_ESM.docx]

**Supplementary materials and methods**

**Microglial purification**

The microglia were purified as previously described [1]. We purified CD11b-positive microglia from the cortex of C57BL/6J group-housed (GH) and juvenile social isolation (j-SI) mice, as well as transgenic mice, at postnatal day 35 (p35; C57BL/6J mice only) and from p58 onward. We purified CD11b-positive microglia from four mouse medial prefrontal cortexes (mPFCs) each in the GH and j-SI groups (mixed because the mPFC from one mouse was too small to purify microglia) to assess MG-*Bdnf* expression in the mPFC. We performed CD11b-positive microglia purification using a magnetic-activated cell sorting (MACS) system. First, mice were fully anesthetized with isoflurane and perfused transcardially with phosphate-buffered saline (PBS). The tissues were then dissociated into single-cell suspensions using the Neural Tissue Dissociation kit (Miltenyi Biotec, Bergisch Gladbach, Germany). Myelin debris was magnetically removed from the single-cell suspensions with Myelin Removal Beads II (Miltenyi Biotec). Next, CD11b-positive microglia were magnetically separated using anti-CD11b microbeads (Miltenyi Biotec). We used the QuadroMACS Separator with LS column (Miltenyi Biotec) for microglia separation and followed the manufacturer’s instructions for all procedures.

**Amplification of cDNA from RNA for quantitative real-time polymerase chain reaction (qRT-PCR)**

In GH and j-SI mice, microglia were purified from the cortex and the mPFC to measure MG-*Bdnf* expression. The amount of mPFC microglia from a single mouse was small; therefore, we purified microglia from the combined mPFC tissues of four GH or j-SI mice. Total RNA was purified from the microglia recovered from the mPFC, and cDNA was amplified from total RNA using a CellAmp™ Whole Transcriptome Amplification Kit (Real Time) Ver. 2 (Takara Bio Inc., Shiga, Japan), according to the manufacturer’s protocol. Due to its relatively large quantity, amplifying the RNA purified from cortex-derived microglia before qRT-PCR was unnecessary.

**Murine peripheral blood mononuclear cell (PBMC) samples** 

Mouse PBMCs were collected as previously described [1]. Whole-blood samples were collected via transcardial perfusion with PBS from GH mice starting from p58 before microglial purification. PBMCs were isolated using Lympholyte-Mammal (Cedarlane Laboratories Ltd., Burlington, Canada) after sampling, and all procedures were conducted following the manufacturer’s instructions.

**qRT-PCR**

In mice, *Bdnf* expression levels were measured in cortical microglia, mPFC microglia (C57BL/6J mice only), the whole cortex, the whole mPFC, and PBMCs at p35 (p35; C57BL/6J mice cortical microglia only) and from p58 onward using qRT-PCR. Total RNA was extracted from the microglia and whole tissues using the Direct-zol™ RNA Microprep kit (Zymo Research, Irvine, CA, USA) or the AllPrep DNA/RNA/Protein Mini Kit (Qiagen, Hilden, Germany), according to the manufacturer’s protocol. Reverse transcription was performed using an iScript kit (Bio-Rad Laboratories, Hercules, CA, USA). qRT-PCR was conducted using SYBR Premix Ex Taq II (Tli RNaseH Plus, Takara Bio Inc.) with a StepOne Plus real-time PCR system and QuantStudio 6 system (Applied Biosystems, Thermo Fisher Scientific Inc., Waltham, MA, USA). For the relative quantification of *Bdnf* expression, *18s* ribosomal RNA was quantified as the housekeeping gene. The primers used were as follows:

*18s* forward: 5′-CGGCTACCACATCCAAGGAA-3′

*18s* reverse: 5′-GCTGGAATTACCGCGGCT-3′ 

*Bdnf* forward: 5′- GCGGCAGATAAAAAGACTGC-3′

*Bdnf* reverse: 5′-CTTATGAATCGCCAGCCAAT-3′ 

We measured complement system-related gene expression in the cortical microglia (transgenic adult mice without doxycycline administration) and the entire mPFC (transgenic adult mice with doxycycline administration starting from p45–p50) using qRT-PCR with the following primers.

*C1qa* forward: 5′-AAAGGCAATCCAGGCAATATCA-3′

*C1qa* reverse: 5′-TGGTTCTGGTATGGACTCTCC-3′

*C1qb* forward: 5′-AAGATCCAGAAACACAAGTCCCT-3′

*C1qb* reverse: 5′-CCTCCTCACCATCAAATGTTGG-3′

*C1qc* forward: 5′-ACACATCGCATACGGCCAA-3′

*C1qc* reverse: 5′-AACATGTGGTCGCAGAAGCTG-3′

*C3ar1* forward: 5′-TCGATGCTGACACCAATTCAA-3′

*C3ar1* reverse: 5′-TCCCAATAGACAAGTGAGACCAA-3′

To evaluate *Bdnf* expression in mPFC microglia, cDNA was amplified from mPFC microglial RNA recovered from four mice, followed by qRT-PCR. The relative quantification of *Bdnf* expression was performed using β-actin (*Actb*) as the housekeeping gene. qRT-PCR from the amplified cDNA was performed using the following primers:

*Actb* forward: 5′-CATCCGTAAAGACCTCTATGCCAAC-3′

*Actb* reverse: 5′-ATGGAGCCACCGATCCACA-3′

*Bdnf* forward: 5′-TCAAGTTGGAAGCCTGAATGAATG-3′

*Bdnf* reverse: 5′-CTGATGCTCAGGAACCCAGGA-3′

*BDNF* mRNA expression in human macrophages was measured using similar methods as in mice, except that the Direct-zol™ RNA Miniprep (ZYMO RESEARCH, Irvine, CA, USA) was used for the RNA purification. The relative quantification of *BDNF* expression was performed using the constitutively expressed genes β-actin (*ACTB*) and cyclophilin A (*CyA*) as internal controls. The following primers were used:

*BDNF* forward: 5′-CATCCGAGGACAAGGTGGCTTGG-3′ 

*BDNF* reverse: 5′-GTCCTCATCCAACAGCTCTTCTATC-3′ 

*ACTB* forward: 5′-GATGTGGATCAGCAAGCA-3′

*ACTB* reverse: 5′-AGAAAGGGTGTAACGCAACTA-3′

*CyA* forward: 5′-GCAGACAAGGTCCCAAAG-3′

*CyA* reverse: 5′-GAAGTCACCACCCTGACAC-3′

**Enzyme-linked immunosorbent assay (ELISA)**

We used ELISA to quantify microglia-derived BDNF in transgenic mice. Microglia purified using the MACS system were washed three times with PBS and dissolved in radioimmunoprecipitation assay buffer (FUJIFILM Wako Pure Chemical Corporation, Osaka, Japan) with a protease inhibitor (Takara Bio Inc.). Proteins were extracted via sonication (Bioruptor, Cosmo Bio Co. Ltd, Tokyo, Japan) and centrifuged, and the supernatant was collected for ELISA. Next, the BDNF concentration was measured using the Mature BDNF ELISA Kit Wako, High Sensitive (FUJIFILM Wako Pure Chemical Corporation) according to the manufacturer’s protocol. Total protein was measured using the Pierce Bicinchoninic Acid Protein Assay kit (Thermo Fisher Scientific Inc.). BDNF concentrations were compared by calculating BDNF/total protein (pg/µg) ratios to correct for the microglia amount purified in each sample. 

**Behavioral tests**

**Three-chamber social preference test**

This test was conducted in an experimental cage (Ohara & Co., Ltd., Tokyo, Japan) comprising a three-chamber rectangular structure with gray walls, floors, and transparent room separators. The day before the test, mice were habituated for 10 min in the central chamber, followed by 10 min in the entire chamber. On the test day, mice were placed in the central chamber for 5 min and allowed to explore the entire chamber for 5 min. Following this exploration, age- and sex-matched C57BL/6J mice were placed in the corral of one chamber. A novel black cube was placed in the corral of the opposite chamber; the subject mice could explore the entire chamber freely during the 10 min test period. Mouse behavior during the test period was evaluated using Any-maze software v.6.34 (Stoelting Co., Wood Dale, IL, USA). Social and object interaction times were calculated as the cumulative time that the mouse was detected in the interaction zone (a circular zone surrounding the corral containing the mouse or object). The social interaction score was defined as follows: (social interaction time − object interaction time) / (social interaction time + object interaction time). All behavioral experiments were started at p56.

**Augmented reality-based long-term animal behavior observing system**

We applied our developed assay system to analyze animal social behavior in a novel environment; this system can determine the position of each mouse under social housing conditions. At least 1 week before the behavioral experiments, we attached an identification (ID) tag (printed with ArUco Markers) to the back of each mouse under anesthesia (400 mg/kg chloral hydrate, intraperitoneally) using an elastic string. The ID tags were treated with quinine to prevent them from being bitten by cagemates. In the behavioral experiment, one subject mouse and three age- and sex-matched C57BL/6J mice that had never been cohoused with the subject mouse were simultaneously caged (276 × 445 × 204 mm; CL-0128; CLEA Japan Inc., Tokyo, Japan) and allowed to move freely for 1 h (Fig. 2e). During the 1 h, the behaviors of all four mice were monitored with an infrared camera at 20 frames/s under infrared illumination. The captured video was analyzed offline using our developed system [2] (now available at Ohara & Co., Ltd.). The central position of the ID tag of each mouse was detected for each frame as XY coordinates within the cage. The time-series coordinate data were exported as comma-separated value (.csv) files. Subsequent data processing was performed using R (version 4.2.1) [3]. We excluded coordinates beyond 1.1 times the estimated floor area of the cage. Additionally, we removed outliers with Z-values exceeding 3, which were calculated for the 2 s period (40 frames) before and after, in the time series, to eliminate misidentified ID tag positions. Coordinates missing in a given frame were linearly interpolated between the previous and next detection coordinates.

Locomotor activity was measured as the distance (in meters) that the ID tag of each mouse moved in the cage within a given period. To analyze social interactions between mice, we defined a contact event when the coordinates of the ID tags of two mice were close by > 20 mm. When two mice made contact, we compared the distances that each mouse moved in the previous second (20 frames). We regarded the mouse that moved a longer distance as the approaching mouse and the other as the receiving mouse. We then defined the number of approaches made and received as the total number of times, in a given period, that a mouse contacted another mouse in the role of the approaching and receiving mouse, respectively.

**Open field test**

The open field test was performed to assess anxiety-like behaviors and activity levels. A gray square acrylic arena (40 × 40 × 40 cm) was used. The subject mice were allowed to explore the arena freely for 30 min. The time spent in the center and the total distance traveled in 30 min were calculated using Any-maze software v.6.34.

**Electrophysiology**

Brain slices containing the mPFC or posterior paraventricular nucleus of thalamus (pPVT) were prepared from mice starting from p61. First, the mice were deeply anesthetized with isoflurane and decapitated. The brain was then quickly removed and immersed in an ice-cold sucrose-based solution (approximately 4 °C) bubbled with a gas mixture of 95% O_2_/5% CO_2_, containing the following (in mM): 210.3 sucrose, 2.5 KCl, 26.2 NaHCO_3_, 1 NaH_2_PO_4_, 0.5 CaCl_2_, 4 MgSO_4_, and 11 D-glucose. The frontal cortexes or brain regions including the pPVT were sectioned into 300 μm-thick slices in the coronal plane using a vibrating tissue slicer (Vibratome 1000 Plus 102, Pelco International, Redding, CA, USA). Slices were incubated for at least 60 min in a chamber filled with standard artificial cerebrospinal fluid (ACSF) continuously bubbled with a gas mixture containing the following (in mM) at 32 °C: 119 NaCl, 2.5 KCl, 26.2 NaHCO_3_, 1 NaH_2_PO_4_, 2 CaCl_2_, 2 MgCl_2_, and 11 D-glucose; the slices were then maintained in the ACSF at 25 °C. Following this incubation, each slice was transferred to a recording chamber. The submerged slice was then superfused at a 2 mL/min flow rate with ACSF saturated with the mixed gas at 32 °C.

Electrophysiological data were recorded from pyramidal cells in layer V of the mPFC (in the prelimbic and infralimbic cortices) or pyramidal cells in the pPVT. Each pyramidal cell was identified using its morphological features (such as large pyramidal or oval cell bodies and thick apical dendrites). Slices were visualized on video images using an upright microscope (BW50WI, Olympus, Tokyo, Japan) with infrared differential interference contrast optics for recording pyramidal cells in the mPFC and using an upright microscope (DM6000FS, Leica) for recording pyramidal cells in the pPVT. 

Cells were current- and voltage-clamped in a conventional whole-cell configuration using a Multiclamp 700A amplifier (Axon Instruments, Molecular Devices, San Jose, CA, USA). Patch pipettes were pulled from borosilicate glass. For current-clamp recordings, pipettes were filled with a low-chloride intracellular solution containing the following (in mM): 127.5 K-methanesulfonate, 5 KCl, 2 MgCl_2_, 2 Mg-ATP, 0.3 Na-GTP, 0.6 ethylene glycol tetraacetic acid (EGTA), and 10 4-(2-hydroxyethyl)-1-piperazineethanesulfonic acid (HEPES); pH 7.25 (adjusted with KOH). For voltage-clamp recordings, we used an intracellular solution containing the following (in mM): 130 Cs-methanesulfonate, 8 NaCl, 4 Mg-ATP, 0.4 Na-GTP, 0.5 EGTA, 10 HEPES, 1.0 QX-314, and 10 Na-phosphocreatine; pH 7.25 (adjusted with CsOH). All membrane potentials were corrected for the liquid junction potential (10 mV for Cs pipette solutions) measured according to a previously established method [4]. Data acquisition and stimulation were controlled using Signal 4 software with Power 1401 interface equipment (Cambridge Electronic Design, Cambridge, UK).

**Current-clamp recordings**

For the current-clamp recordings, series resistance was monitored and canceled using a bridge circuit, and pipette capacitance was compensated. Voltage signals were low-pass filtered at 10 kHz and digitized at 20 kHz. The baseline membrane potential was maintained near −70 mV with the current injection. We recorded membrane potential responses to hyperpolarizing and depolarizing current pulses (500 ms in duration) to examine action potentials and subthreshold membrane properties. Depolarizing current pulses with 10–200 PA intensities were injected at 10 pA increments. 

**Voltage-clamp recordings**

Regarding the voltage-clamp recordings, pipette capacitance was compensated, whereas series resistance was continuously monitored and not compensated—only recordings with a stable series resistance < 20 MΩ were used for the analyses. Current signals were low-pass filtered at 800 Hz and digitized at a sampling frequency of 10 kHz. Spontaneous excitatory postsynaptic currents (sEPSCs) and spontaneous inhibitory postsynaptic currents (sIPSCs) were recorded in standard ACSF. Miniature EPSCs (mEPSCs) and miniature IPSCs (mIPSCs) were recorded in standard ACSF with 1.0 µM tetrodotoxin. EPSCs and IPSCs were separated by holding the neuron at the reversal potential for excitatory or inhibitory postsynaptic currents, allowing for EPSC and IPSC isolation at −70 mV and 0 mV, respectively.

**Data analysis** 

We analyzed the membrane potential data obtained from the current-clamp recordings using Signal 4 software and evaluated each cell’s intrinsic membrane and action potential. The input resistance of neurons was estimated using a linear regression coefficient for changes in peak voltage caused by injected hyperpolarizing currents (−50 pA to −10 pA, 500 ms). The rheobase current threshold was the minimum current at which the injected current elicited at least an action potential. For the action potential elicited by the rheobase current injection, the action potential threshold was measured using the derivatives of the voltage curve. The action potential threshold was the voltage at which the slope of the action potential traced was 10 mV/ms. The spike amplitude was the voltage from the threshold to the peak of the action potential at a 200 pA current injection from the baseline. We used Mini Analysis software (Synaptosoft, Fort Lee, NJ, USA) to detect and analyze sEPSCs, mEPSCs, sIPSCs, and mIPSCs in the membrane current data obtained from voltage-clamp recordings. For each cell, all PSCs were detected for 2.5 min, and the mean amplitude and frequency were calculated. 

**Whole-cell RNA sequencing (RNA-seq)**

The mPFCs were recovered from *Iba1*-tTA(+)::*Bdnf*^(tetO/+)^ and *Bdnf*^(tetO/+)^ mice starting from p63 and analyzed using total RNA-seq. Each mouse was anesthetized entirely with isoflurane and perfused transcardially with PBS before the brain was removed, and the mPFC was collected using a brain slicer. For cultured cells, total RNA was extracted using the Direct-zol RNA MicroPrep kit (Zymo Research, Irvine, CA, USA), according to the manufacturer’s protocol. The total RNA was sent to DNA Chip Research Inc. (Yokohama, Japan), where RNA-seq was performed. The total RNA obtained from each sample was subjected to sequencing library construction using the TruSeq Stranded mRNA Library Prep Kit (Illumina, San Diego, CA, USA), according to the manufacturer’s instructions. Library quality was assessed using an Agilent Technologies 2100 Bioanalyzer (Agilent Technologies, Santa Clara, CA, USA). Pooled library samples were sequenced on a NovaSeq 6000 instrument (Illumina) with 100 bp paired-end reads. Sequencing adaptors, low-quality reads, and bases were trimmed with the Trimmomatic-0.39 tool [5]. The sequence reads were aligned to the mouse reference genome (mm10) using STAR ver. 2.7.9a [6]. Aligned reads were subjected to downstream analysis using StrandNGS v4.0 software (Agilent Technologies). The number of reads for each gene and transcript was quantified using the transcripts per million method [7, 8]. We set the statistical cutoff for differentially expressed genes as a fold-change (log_2_) ≥ 1.5 or ≤ −1.5. The significance of the analysis was defined as p < 0.05 based on the Benjamini–Hochberg-corrected p-value [9]. Gene Ontology analysis was performed based on molecular functions using StrandNGS v4.0 software, with p-values of enrichment determined using a hypergeometric distribution test adjusted for multiple tests with the Benjamini–Hochberg procedure. Principal component analysis, hierarchical clustering analysis, volcano plot generation, and heatmap generation with the Z-score were performed using StrandNGS v4.0 software. Principal component analysis coordinates were plotted onto XYZ axes using R (version 4.2.1) [3]. The genes in the Wnt signaling pathway were those listed in the Kyoto Encyclopedia of Genes and Genomes “Wnt signaling pathway – Musculus” [10].

**Human macrophage samples and assessment of childhood experiences**

The studies involving human participants were reviewed and approved by the Nara Medical University Ethics Committee (Approval Number: 1319). The patients/participants provided their written informed consent to participate in this study. We included 21 patients with autism spectrum disorder (ASD; age: 30.10 ± 6.457 years, five females) and 18 typically developing individuals (age: 29.44 ± 6.546 years, six females). All participants were Japanese and were born and lived in Japan. Patients with ASD were recruited from the outpatient service of the Department of Psychiatry, Nara Medical University Hospital, and its affiliated psychiatric clinic. Two trained psychiatrists diagnosed ASD using the Diagnostic and Statistical Manual 5 (DSM-5) criteria, which were further validated by trained psychiatrists and staff using the Autism Diagnostic Observation Schedule-2 (ADOS-2) [11]. Each participant’s full intelligence quotient (FIQ) was estimated using the similarity and symbol search subtests of the Wechsler Adult Intelligence Scale, Third Edition [12]. 

The following exclusion criteria were used: participants < 17 years; participants with low intelligence (FIQ < 70); participants diagnosed with other neurological and psychiatric disorders, as assessed during the Mini International Neuropsychiatric Interview; participants using steroids. All participants completed a self-reported measure to assess adverse childhood experiences: the Japanese version of the Child Abuse and Trauma Scale (CATS) [13], a 38-item instrument that retrospectively evaluates adverse childhood experiences [14]. Each item is measured on a five-point scale of 0–4 and is divided into five major factors of adverse childhood experiences: neglect or negative home atmosphere, sexual abuse, punishment, emotional abuse, and others. The total score was the sum of the scores.

Monocyte isolation and macrophage differentiation were performed as previously described [15]. Monocytes were isolated using MACS and the CellXVivo Human M1 or M2 Macrophage Differentiation Kit (R&D Systems, Minneapolis, MN, USA), according to the manufacturer’s protocol. Briefly, whole human blood samples were collected through venipuncture during the daytime (09:00–16:00) and stored on ice. PBMCs were immediately separated from whole blood via density-gradient centrifugation using the separation medium Lymphoprep (Serumwerk Bernburg AG, Bernburg, Germany), separation tubes, and Leucosep (Greiner Bio-One, Kremsmünster, Austria). CD14-positive monocytes were isolated from PBMCs using a MACS system with CD14 microbeads (Miltenyi Biotec). 

CD14-positive monocytes were resuspended in PBS containing 0.5% bovine serum albumin (Sigma-Aldrich, St. Louis, MO, USA), 2 mM ethylenediaminetetraacetic acid, and a 1% penicillin–streptomycin mixed solution (Nacalai Tesque, Kyoto, Japan). The cells were seeded at a 1 × 10^6^ cells/mL density onto M1 or M2 differentiation medium containing recombinant human granulocyte-macrophage colony-stimulating factor or recombinant human macrophage colony-stimulating factor, respectively, at 37 °C in a humified atmosphere of 5% CO_2_. We replaced half of each culture with fresh medium on day 3 and obtained M1 or M2 macrophages on day 6.

**Supplementary references**

1. Ikawa D, Makinodan M, Iwata K, Ohgidani M, Kato TA, Yamashita Y et al. Microglia-derived neuregulin expression in psychiatric disorders. *Brain Behav Immun* 2017; **61**: 375–385.

2. Endo N, Makinodan M, Mannari-Sasagawa T, Horii-Hayashi N, Somayama N, Komori T et al. The effects of maternal separation on behaviours under social-housing environments in adult male C57BL/6 mice. *Sci Rep* 2021; **11**: 527.

3. R Core Team. *R: A language and environment for statistical computing*. R Foundation for Statistical Computing: Vienna, Austria, 2022.

4. Neher E. Correction for liquid junction potentials in patch clamp experiments. *Methods Enzymol* 1992; **207**: 123–131.

5. Bolger AM, Lohse M, Usadel B. Trimmomatic: a flexible trimmer for Illumina sequence data. *Bioinformatics* 2014; **30**: 2114–2120.

6. Dobin A, Davis CA, Schlesinger F, Drenkow J, Zaleski C, Jha S et al. STAR: ultrafast universal RNA-seq aligner. *Bioinformatics* 2013; **29**: 15–21.

7. Wagner GP, Kin K, Lynch VJ. Measurement of mRNA abundance using RNA-seq data: RPKM measure is inconsistent among samples. *Theory Biosci* 2012; **131**: 281–285.

8. Li B, Dewey CN. RSEM: accurate transcript quantification from RNA-Seq data with or without a reference genome. *BMC Bioinformatics* 2011; **12**: 323.

9. Benjamini Y, Drai D, Elmer G, Kafkafi N, Golani I. Controlling the false discovery rate in behavior genetics research. *Behav Brain Res* 2001; **125**: 279–284.

10. Kanehisa M, Goto S. KEGG: Kyoto Encyclopedia of Genes and Genomes. *Nucleic Acids Res* 2000; **28**: 27–30.

11. Lord C, Rutter M, Goode S, Heemsbergen J, Jordan H, Mawhood L et al. Autism diagnostic observation schedule: a standardized observation of communicative and social behavior. *J Autism Dev Disord* 1989; **19**: 185–212.

12. Sumiyoshi C, Fujino H, Sumiyoshi T, Yasuda Y, Yamamori H, Ohi K et al. Usefulness of the Wechsler Intelligence Scale short form for assessing functional outcomes in patients with schizophrenia. *Psychiatry Res* 2016; **245**: 371–378.

13. Tanabe H, Ozawa S, Goto K. Psychometric properties of the Japanese version of the Child Abuse and Trauma Scale (CATS). *The 9th Annual Meeting of the Japanese Society for Traumatic Stress Studies*. 2010.

14. Sanders B, Becker-Lausen E. The measurement of psychological maltreatment: early data on the Child Abuse and Trauma Scale. *Child Abuse Negl* 1995; **19**: 315–323.

15. Yamauchi T, Makinodan M, Toritsuka M, Okumura K, Kayashima Y, Ishida R et al. Tumor necrosis factor-α expression aberration of M1/M2 macrophages in adult high-functioning autism spectrum disorder. *Autism Res* 2021; **14**: 233–2341.

**Supplementary figure legends**

**Supplementary Fig. 1: Open field test behaviors or** ***Bdnf*** **mRNA expression between group-housed (GH) and juvenile social isolation (j-SI) mice in whole tissue regions.**

(a) Schema of the GH and j-SI mice time series. The experiments were started at postnatal day 59 (p59). (b) No differences were observed in the basal activity or time spent in the center between j-SI and GH mice in the open field test. (Left) Distance traveled (U = 63, p = 0.4371, Mann–Whitney *U* test, GH: n = 12, j-SI: n = 13). (Right) Time at the center (U = 73, p = 0.8100, Mann–Whitney *U* test, GH: n = 12, j-SI: n = 13). (c) No differences were observed in *Bdnf* mRNA expression, measured using RT-qPCR, in the whole cortex or whole medial prefrontal cortexes (mPFCs) between GH and j-SI mice. (Left) *Bdnf* mRNA expression in the whole cortex (t_(6) _= 0.9354, p = 0.3857, unpaired two-tailed Student’s *t*-test, GH: n = 4, j-SI: n = 4). (Right) *Bdnf* mRNA expression in the whole mPFC ((t_(6) _= 0.1454, p = 0.8892, unpaired two-tailed Student’s *t*-test, GH: n = 4, j-SI: n = 4). Data are presented as the mean ± SEM. 2M: two months of age, A.U.: arbitrary unit.

**Supplementary Fig. 2: Overexpressed MG-*****Bdnf*** **does not alter test behaviors, increase** ***Bdnf*** **mRNA expression levels in whole tissue regions, or affect the electrical properties of posterior paraventricular nucleus of thalamus (pPVT) neurons.**

(a) The experiments were started at postnatal day 65 (p65) without doxycycline treatment. (b) No differences were observed in *Bdnf* mRNA expression, measured using RT-qPCR, in the whole cortex or medial prefrontal cortex (mPFC) between Iba1-BDNF and control mice. (Left) *Bdnf* mRNA expression in the whole cortex (t_(5) _= 0.5269, p = 0.6208, unpaired two-tailed Student’s *t*-test, control: n = 4, Iba1-BDNF: n = 3). (Right) *Bdnf* mRNA expression in the whole mPFC (t_(6) _= 0.6909, p = 0.5155, unpaired two-tailed Student’s *t*-test, control: n = 4, Iba1-BDNF: n = 4). (c) No differences were observed in basal activity or time spent in the center between the Iba1-BDNF and control mice in the open field test. (Left) Distance traveled (U = 65, p = 0.7125, Mann–Whitney *U* test, control: n = 12, Iba1-BDNF: n = 12). (Right) Time at the center (U = 56, p = 0.3777, Mann–Whitney *U* test, control: n = 12, Iba1-BDNF: n = 12). (d) Representative traces of spontaneous excitatory postsynaptic currents (sEPSCs) onto pPVT neurons. (e) There was no significant change in both (left) the sEPSC frequency (t_(24) _= 0.08757, p = 0.9309, unpaired two-tailed Student’s *t*-test) and (right) the sEPSC amplitude (U = 67, p = 0.3897, Mann–Whitney U test) between the control and Iba1-BDNF mice. (f) Representative traces of spontaneous inhibitory postsynaptic currents (sIPSCs) onto pPVT neurons. (g) There was no significant change in both (left) the sIPSC frequency (U = 55, p = 0.1389, Mann–Whitney U test) and (right) the sIPSC amplitude (U = 57, p = 0.1690, Mann–Whitney U test) between the control and Iba1-BDNF mice. (e, g) n = 13 cells from three biologically independent control mice, n = 13 cells from three biologically independent Iba1-BDNF mice. Data are presented as the mean ± SEM. 2M: two months of age, A.U.: arbitrary unit, control: *Bdnf*^(tetO/+) ^mice, Iba1-BDNF: *Iba1*-tTA(+)::*Bdnf*^(tetO/+)^ mice.

**Supplementary Fig. 3: Overexpressed MG-BDNF** **affects the Wnt signaling pathway.**

(a) Heatmap of Wnt signaling pathway-related genes with reference to the “Wnt signaling pathway” in the Kyoto Encyclopedia of Genes and Genomes (KEGG) database. (b) Heatmap of neurotrophic factor and cytokine genes. (c) No significant differences in *C1qa* (t_(6) _= 0.8411, p = 0.4325, unpaired two-tailed Student’s *t*-test), *C1qb* (t_(6) _= 0.7014, p = 0.5093, unpaired two-tailed Student’s *t*-test), *C1qc* (U = 7, p = 0.8857, Mann–Whitney *U* test), and *C3ar1* (U = 7, p = 0.8857, Mann–Whitney *U* test) mRNA expression were found in cortex microglia between Iba1-BDNF and control mice (control: n = 4, Iba1-BDNF: n = 4). Data are presented as the mean ± SEM. Control: *Bdnf*^(tetO/+) ^mice, Iba1-BDNF: *Iba1*-tTA(+)::*Bdnf*^(tetO/+)^ mice.

**Supplementary Fig. 4: MG-BDNF normalization from the juvenile period does not affect activity levels or time spent in the center.**

Doxycycline administration from postnatal day 21 (p21) did not affect the activity or time spent in the center in the open field test. The open field test was started at p59. (Left) Distance traveled (U = 63, p = 0.4371, Mann–Whitney *U* test, control: n = 12, Iba1-BDNF: n = 13). (Right) Time at the center (t_(23) _= 0.3380, p = 0.7384, unpaired two-tailed Student’s *t*-test, control: n = 12, Iba1-BDNF: n = 13). Data are presented as the mean ± SEM. 2M: two months of age, control: *Bdnf*^(tetO/+) ^mice, Iba1-BDNF: *Iba1*-tTA(+)::*Bdnf*^(tetO/+)^ mice.

**Supplementary Fig. 5: Normalizing MG-BDNF during adulthood results in long-lasting inhibitory input abnormalities in the medial prefrontal cortex (mPFC).**

(a–h) Iba1-BDNF mice were administered doxycycline from postnatal day 45–50 (p45–p50) to normalize MG-BDNF. Control mice were also administered doxycycline from p45–p50. The experiments were started at p60. (b) Normalizing MG-BDNF in Iba1-BDNF mice from p45–p50 eliminated the difference in the three-chamber social test. The two groups showed no differences in the social interaction score (U = 214, p = 0.3857, Mann–Whitney *U* test, control: n = 22, Iba1-BDNF: n = 23) or social investigation time (F_1,43 _(interaction) = 0.04286, p = 0.8370, two-way ANOVA, control: n = 22, Iba1-BDNF: n = 23). S, social; O, object. (c) Doxycycline administration from p45–p50 did not affect the activity or time spent in the center in the open field test. (Left) Distance traveled (t_(23) _= 1.064, p = 0.2983, unpaired two-tailed Student’s *t*-test, control: n = 9, Iba1-BDNF: n = 16. (Right) Time at the center (t_(23) _= 0.6348, p = 0.5318, unpaired two-tailed Student’s *t*-test, control: n = 9, Iba1-BDNF: n = 16). (d) The spike frequency remained reduced in Iba1-BDNF mice even after MG-BDNF was restored from p45–p50 (left) (U = 68.50, p = 0.0133, Mann–Whitney *U* test). No differences were observed in the spike amplitude (middle) (U = 130, p = 0.8451, Mann–Whitney *U* test) or threshold (right) (U = 101, p = 0.2171, Mann–Whitney *U* test) (n = 16 cells from three biologically independent control mice, n = 17 cells from four biologically independent Iba1-BDNF mice). (e) No differences in the spontaneous excitatory postsynaptic current (sEPSC) frequency were observed between the control and Iba1-BDNF mice treated with doxycycline from p45–p50 (U = 129, p = 0.3359, Mann–Whitney *U* test) or amplitude (U = 143, p = 0.6037, Mann–Whitney *U* test). (f) The spontaneous inhibitory postsynaptic currents (sIPSC) frequency remained increased in Iba1-BDNF mice even after MG-BDNF was restored from p45–p50 (U = 37, p < 0.0001, Mann–Whitney *U* test). Control mice had higher sIPSC amplitudes than Iba1-BDNF mice treated with doxycycline from p45–p50 (U = 58, p = 0.0008, Mann–Whitney *U* test). (e, f) n = 16 cells from four biologically independent control mice, n = 20 cells from four biologically independent Iba1-BDNF mice. (g) No differences in the miniature EPSC (mEPSC) frequency were observed between the control and Iba1-BDNF mice treated with doxycycline from p45–p50 (U = 86, p = 0.4488, Mann–Whitney *U* test) or amplitude (t_(27) _= 0.8768, p = 0.3883, unpaired two-tailed Student’s *t*-test). (h) mIPSC frequency remained increased in Iba1-BDNF mice even after MG-BDNF was restored from p45–p50 (U = 42, p = 0.0056, Mann–Whitney *U* test). No difference was observed in the miniature IPSC (mIPSC) amplitude between the control and Iba1-BDNF mice treated with doxycycline from p45–p50 (U = 66, p = 0.1006, Mann–Whitney *U* test). (g, h) n = 13 cells from four biologically independent control mice, n = 16 cells from four biologically independent Iba1-BDNF mice. (i) No significant differences in *C1qa* (t_(6) _= 0.1660, p = 0.8736, unpaired two-tailed Student’s *t*-test), *C1qb* (t_(6) _= 1.298, p = 0.2420, unpaired two-tailed Student’s *t*-test), *C1qc* (U = 6, p = 0.6857, Mann–Whitney *U* test), and *C3ar1* (t_(6) _= 0.2927, p = 0.7796, unpaired two-tailed Student’s *t*-test) mRNA expression were found in the mPFC between Iba1-BDNF and control mice treated with doxycycline from p45–p50 (control: n = 4, Iba1-BDNF: n = 4). *p  <  0.05, **p  <  0.01, ****p  <  0.0001. Data are presented as the mean ± SEM. 2M: two months of age, control: *Bdnf*^(tetO/+) ^mice, Iba1-BDNF: *Iba1*-tTA(+)::*Bdnf*^(tetO/+)^ mice.

**Supplementary Fig. 6:** ***BDNF*** **expression correlation between the peripheral blood and central microglia** 

(a) A positive correlation was observed between the *Bdnf* expression levels in PBMC and microglia in mice (r = 0.6154, p = 0.0373, Spearman’s rank correlation coefficient, group-housed mice: n = 12). (b) Schematic flow of human macrophage *BDNF* expression measured using RT-qPCR. Monocytes were collected from peripheral blood and differentiated into M1/M2 macrophages. A.U.: arbitrary unit, PBMC: peripheral blood mononuclear cells.

**Supplementary Table 1. Correlation between human macrophage** ***BDNF*** **expression and Child Abuse and Trauma Scale (CATS) sub-item scores**

rs: Spearman’s rank correlation coefficient. 

The false discovery rate was controlled using the Benjamini–Hochberg method to adjust for multiple comparisons; values with q = 0.033 and p < 0.033 were considered significant (*).
